# Supplementary material for: Socioeconomic inequalities in healthcare utilisation in Indonesia: a comprehensive survey-based overview
Source: BMJ Open. 2019 Jul 19;9(7):e026164. doi: 10.1136/bmjopen-2018-026164 (PMC6661624; doi:10.1136/bmjopen-2018-026164)
Supplement: Supplementary data [file bmjopen-2018-026164supp001.pdf]

Table 1. Distribution of self-assessed health status (SAH) among different socioeconomic status (SES)

	Self-assessed health (SAH)			
	Very healthty<br>n (%)	Somewhat healthy<br>n (%)	Somewhat unhealthy<br>n (%)	Very unhealthy<br>n (%)
**Educational level**				
Pre-primary	1900 (19.3)	4922 (49.9)	2693 (27.3)	353 (3.6)
Primary	1980 (19.8)	5652 (56.6)	2178 (21.8)	182 (1.8)
Lower secondary	1538 (19.0)	4956 (61.3)	1499 (18.5)	89 (1.1)
Upper secondary	2044 (19.0)	6957 (64.8)	1641 (15.3)	90 (0.8)
Tertiary	675 (19.8)	2270 (66.6)	436 (12.8)	27 (0.8)
**Income**				
1st quintile (poorest)	1683 (18.8)	5031 (56.3)	2036 (22.8)	194 (2.2)
2nd quintile	1637 (18.9)	5077 (58.5)	1778 (20.5)	188 (2.2)
3rd quintile	1649 (19.4)	5081 (59.8)	1619 (19.1)	143(1.7)
4th quintile	1622 (19.8)	4894 (59.7)	1570 (19.2)	112 (1.4)
5th quintile (richest)	1544 (19.9)	4674 (60.2)	1444 (18.6)	105 (1.4)

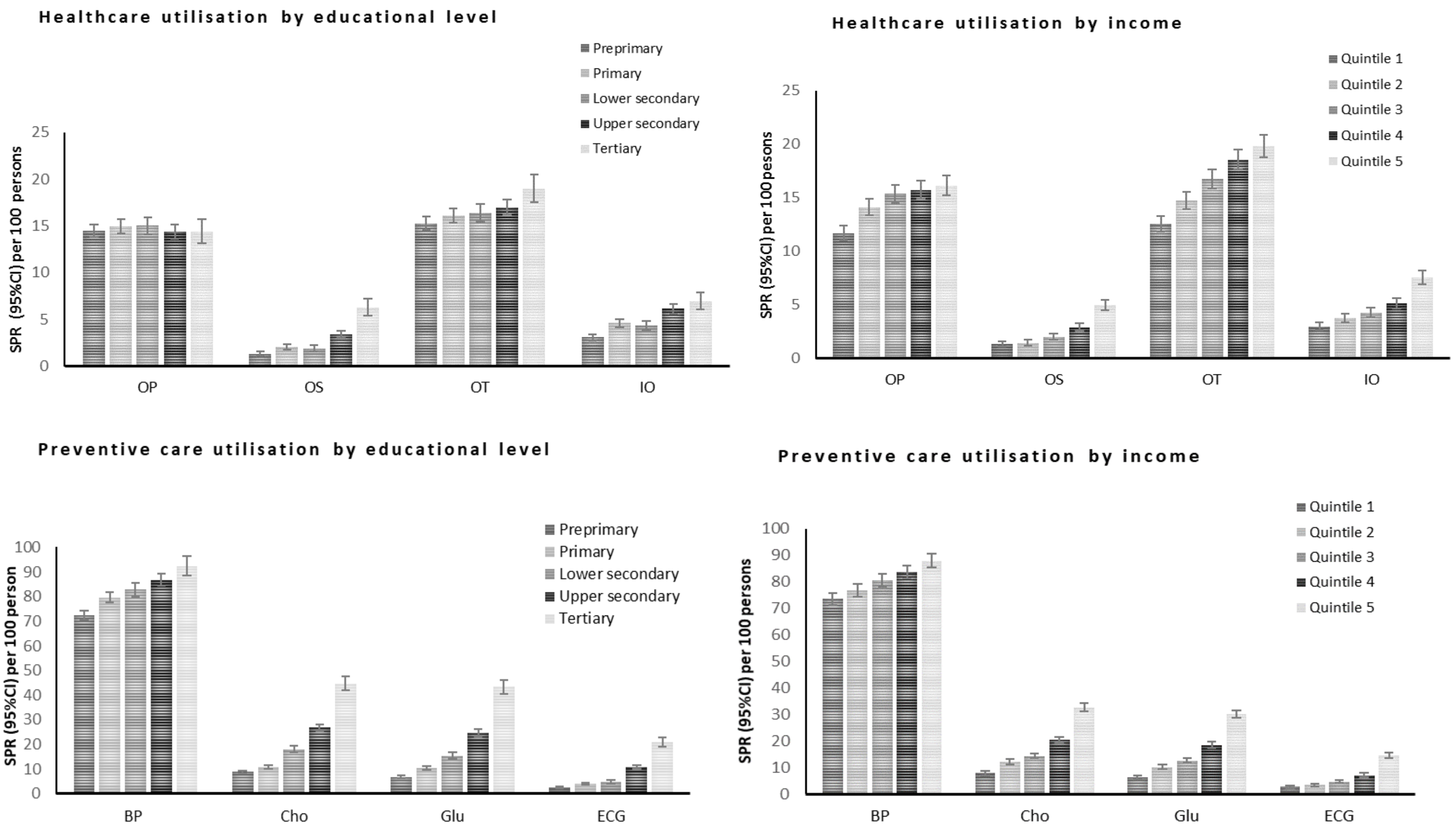

Figure 1. Standardised prevalence rate (95%CI) for healthcare and preventive care utilisation. Prevalence rate is per 100 persons, standardised by age and sex to total population. OP: Outpatient primary care; OS: Outpatient secondary care; OT: Outpatient total; IO: Inpatient overall; BP: Blood pressure; Cho: Cholesterol; Glu: Blood glucose; ECG: Electrocardiograph.

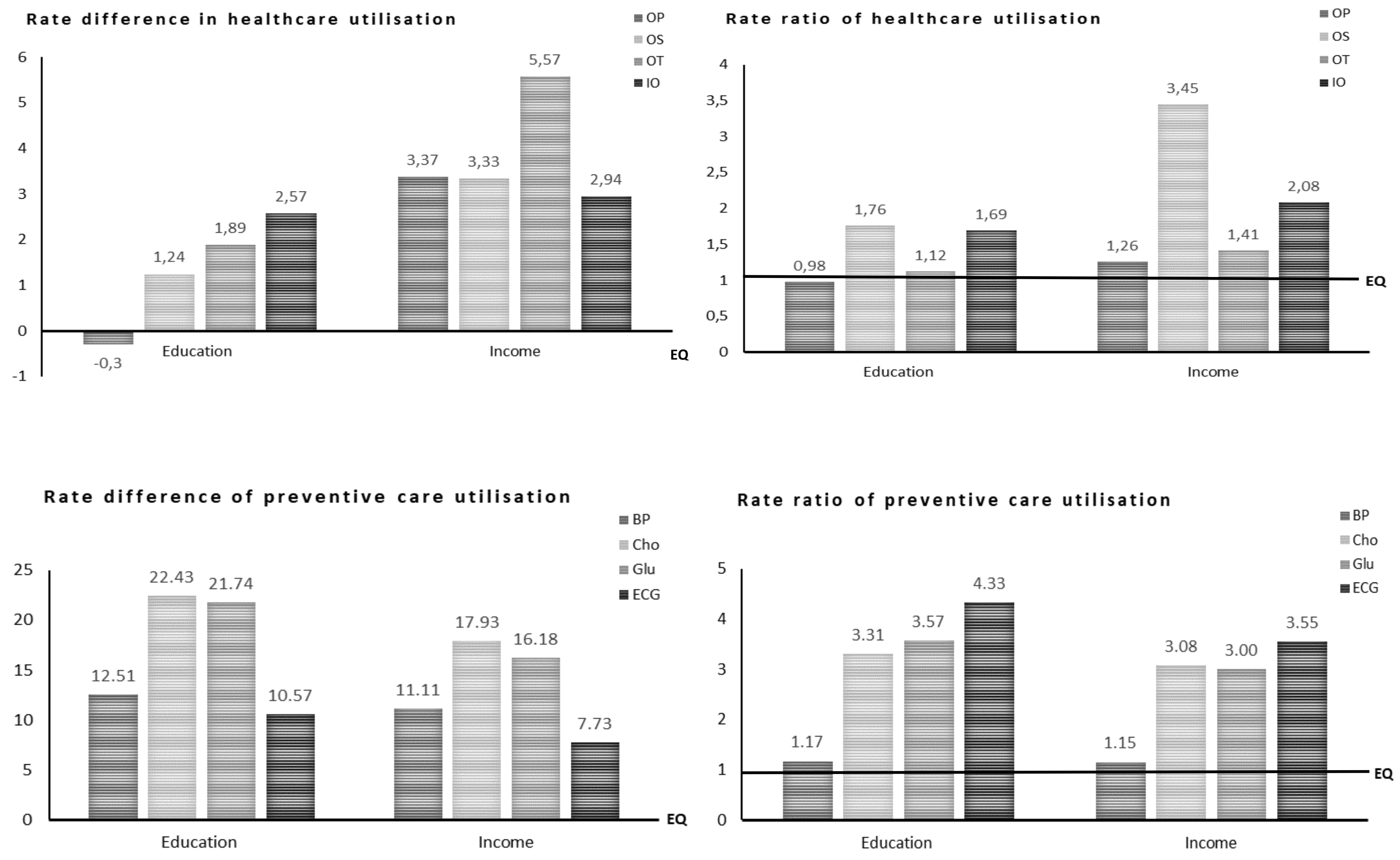

Figure 2. **Simple measurement of absolute (rate difference) and relative (rate ratio) inequalities in healthcare and preventive care utilisation between two highest and two lowest groups of SES. Rate difference is per 100 persons. OP: Outpatient primary care; OS: Outpatient secondary care; OT: Outpatient total; IO: Inpatient overall; BP: Blood pressure; Cho: Cholesterol; Glu: Blood glucose; ECG: Electrocardiograph; EQ: Equality line.**

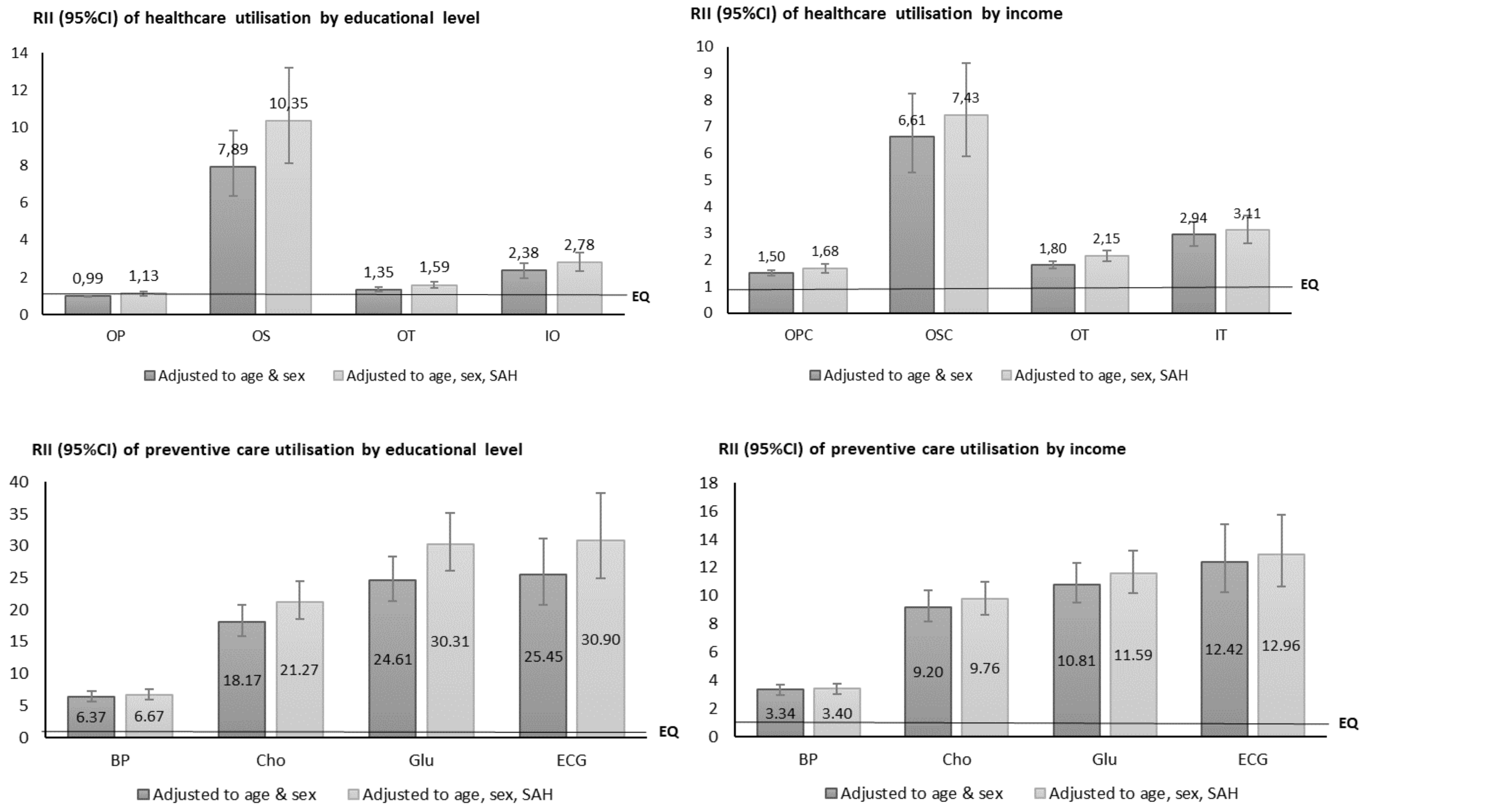

Figure 3. Relative index inequality (95%CI) of healthcare and preventive care utilisation by educational level and income. OP: Outpatient primary care; OS: Outpatient secondary care; OT: Outpatient total; IO: Inpatient overall; BP: Blood pressure; Cho: Cholesterol; Glu: Blood glucose; ECG: Electrocardiograph; EQ: Equality line; SAH: Self-assessed health
