# Supplementary material for: Socioeconomic inequalities in healthcare utilisation in Indonesia: a comprehensive survey-based overview
Source: BMJ Open. 2019 Jul 19;9(7):e026164. doi: 10.1136/bmjopen-2018-026164 (PMC6661624; doi:10.1136/bmjopen-2018-026164)
Supplement: Author's manuscript [file bmjopen-2018-026164.draft_revisions.pdf]

BMJ Open is committed to open peer review. As part of this commitment we make the peer review history of every article we publish publicly available.

When an article is published we post the peer reviewers' comments and the authors' responses online. We also post the versions of the paper that were used during peer review. These are the versions that the peer review comments apply to.

The versions of the paper that follow are the versions that were submitted during the peer review process. They are not the versions of record or the final published versions. They should not be cited or distributed as the published version of this manuscript.

BMJ Open is an open access journal and the full, final, typeset and author-corrected version of record of the manuscript is available on our site with no access controls, subscription charges or pay-per-view fees (http://bmjopen.bmj.com).

If you have any questions on BMJ Open's open peer review process please email info.bmjopen@bmj.com

BMJ Open

Socioeconomic inequalities in healthcare utilisation in Indonesia: a comprehensive survey-based overview

Journal:	*BMJ Open*
Manuscript ID	bmjopen-2018-026164
Article Type:	Research
Date Submitted by the Author:	20-Aug-2018

Complete List of Authors:	Mulyanto, Joko; Amsterdam UMC University of Amsterdam, Public Health; Faculty of Medicine Universitas Jenderal Soedirman, Public Health and Community Medicine<br>Kringos, Dionne Sofia; Amsterdam UMC University of Amsterdam, Public Health<br>Kunst, Anton; Amsterdam UMC University of Amsterdam, Public Health

Keywords:	Socioeconomic, Inequalities, Access, Healthcare, Indonesia

SCHOLARONE™
Manuscripts

Title:

Socioeconomic inequalities in healthcare utilisation in Indonesia: a comprehensive survey-based overview

Authors:

Joko Mulyanto[1,2], Dionne S Kringos[1], Anton E Kunst[1]

[1]Department of Public Health, Amsterdam UMC, University of Amsterdam, Amsterdam Public Health Research Insitute, Amsterdam, the Netherlands.

[2]Department of Public Health and Community Medicine, Faculty of Medicine, Universitas Jenderal Soedirman, Purwokerto, Indonesia

Corresponding author

Joko Mulyanto, Department of Public Health, Amsterdam UMC, University of Amsterdam, PO Box 22660 1100 DD, Amsterdam, the Netherlands; Telephone: +31-20-5664642; j.mulyanto@amc.uva.nl

Word count

Main text: 2969 words

Abstract: 241 words

Tables: 5 tables

For peer review only

ABSTRACT

Objective Monitoring inequality in healthcare utilisation is essential to reduce persistent inequalities in health in lower-middle income countries. This study aimed to assess socioeconomic inequalities in the utilisation of primary care, secondary care, and preventive care in Indonesia.

Methods A cross-sectional study was conducted using data from the 2014 Indonesia Family Life Survey with a total of 42,083 adult participants. Socioeconomic status (SES) was measured by educational level and income. Healthcare utilisation was measured in: i) primary care, ii) outpatient in secondary care, iii) inpatient care, and iii) cardiovascular-related preventive care. The magnitude of inequalities was measured using the relative index of inequality (RII).

Results Small educational inequalities were found for primary care utilisation (RII 1.13, 95% CI 1.01-1.26). Larger educational inequalities were found for outpatient secondary care (RII 10.35, 95% CI 8.11-13.22) and inpatient care (RII 2.78, 95% CI 2.32-3.32). The largest educational inequalities were found for preventive care, particularly regarding blood glucose tests (RII 30.31, 95% CI 26.13-35.15) and electrocardiography tests (RII 30.90, 95% CI 24.97-38.23). Compared to educational inequalities, income inequalities were larger for primary care (RII 1.68, 95% CI 1.52-1.85) and inpatient care (RII 3.11, 95% CI 2.63-3.66), but not for outpatient secondary care and preventive care.

Conclusions Socioeconomic inequalities in healthcare utilisation in Indonesia are particularly large in secondary and preventive care. Therefore, it is recommended to prioritise policies focused on improving timely, geographical and financial access to secondary and preventive care for lower SES groups.

Keywords socieconomic: inequalities; access; healthcare: Indonesia

For peer review only

For peer review only - http://bmjopen.bmj.com/site/about/guidelines.xhtml

ARTICLE SUMMARY

Strengths and limitations of this study

- This study was based on a national representative survey with a high response rate and with measurements that matched established international standards.
- Few studies have investigated inequalities in healthcare utilisation in Indonesia
- The measurement of healthcare need was limited to self-assessed health.
- The measurement of healthcare utilisation was based on self-reported data which might be subject to recall bias.

For peer review only

For peer review only - http://bmjopen.bmj.com/site/about/guidelines.xhtml

INTRODUCTION

Equal use of healthcare for equal need is essential to improve population health, and is therefore an objective for most healthcare systems. Monitoring inequality in healthcare utilisation is essential to assess the performance of a healthcare system, and ultimately, to reduce persistent inequalities in health.[1] To monitor inequality in use in these terms, healthcare utilisation should to be adjusted for self-assessed health or morbidities, as determinants of healthcare need.[2 3]

There is evidence of inequalities in healthcare utilisation in developed countries, despite universal healthcare coverage.[4] For example, both in Western and Eastern Europe, inequalities in healthcare utilisation exist for certain types of healthcare. In Eastern Europe, the rapid transition of the healthcare system since the late 1990s after the fall of Communism may have been conducive to large inequalities.[5 6]

Lower-middle income countries (LMICs) also experience inequalities in healthcare utilisation, and especially in secondary care, as shown by an international comparative study in Asia, Africa and Latin America[7]; and studies in China and India.[8 9] Significant inequalities in healthcare utilisation are also found in Thailand, despite universal healthcare coverage since 2005.[10] For several reasons, sizeable inequalities in healthcare utilisation may also exist in other LMICs. Many of these countries are struggling to provide universal healthcare coverage, resulting in persisting financial barriers to access healthcare. Furthermore, inadequate supply and unequal geographic distribution of healthcare facilities cause greater barriers to the use of these facilities by people living in remote places and with limited resources. Moreover, large inequalities in the quality of healthcare that is actually received may result from poor stewardship, low financial investments in the healthcare system, and suboptimal quality of a broad range of healthcare services.[11 12]

For peer review only - http://bmjopen.bmj.com/site/about/guidelines.xhtml

Indonesia is a LMIC with a population of 262 million people that are distributed across ≥17,000 islands, and with diverse ethnic and religious backgrounds. Indonesia's healthcare system is a mixture of public and private healthcare delivery systems. The size and role of the private-commercial healthcare market has increased during the last decade. Total health expenditure in 2014 was 2.8% of the gross domestic product, of which 47% originated from out-of-pocket payments. Since 1999, the government has provided health insurance for the poor, and in 2014 introduced the National Health Insurance (NHI) program to remove financial barriers to access basic healthcare services for the entire population by 2019.[13] However, over the years, progress towards universal health coverage has been uneven and iterative, and consistently driven by domestic political interests as opposed to technical considerations.[14] This has resulted in, for instance, an inadequate supply and uneven distribution of healthcare resources, which may have contributed to considerable inequalities in healthcare utilisation.[13]

Few studies have investigated inequalities in healthcare utilisation in Indonesia. A study in 2007 showed that lower income groups used significantly less child care and prenatal care services in primary care (PC) than higher income groups.[15] Another study revealed inequalities in dental care utilisation in Indonesia during the period 1999-2009.[16 17] Furthermore, a study conducted in 2011 showed substantial education and income inequality in the utilisation of maternal and child care.[18] A report from the WHO stated that large inequalities in maternal and child healthcare still persist in Indoneisa, in addition to geographic inequalities in the healthcare infrastructure, particularly between the different provinces.[19] A recent survey showed that Indonesian people perceived significant inequalities in the provision of basic services such as healthcare. Interviewees deemed these inequalities to be unjust and expressed the need to address these inequalities.[20]

However, no studies have empirically assessed socioeconomic inequalities in general healthcare utilisation in the adult population in Indonesia. The general aim of the present study is to fill in this gap of evidence. Using a large-scale national interview survey, we aimed to provide a comprehensive

For peer review only - http://bmjopen.bmj.com/site/about/guidelines.xhtml

overview of socioeconomic inequalities in the utilisation of PC, secondary care and preventive care among the adult population in Indonesia.

METHODS

Study design and data sources

Data was used from the fifth wave of the Indonesia Family Life Survey (IFLS5) which was conducted in 2014 by the RAND Corporation (USA). The data are publicly accessible through RAND's website.[21] The IFLS5 was approved by the relevant ethical review committees in the USA and Indonesia. It collected data from 13 Indonesian provinces that together represent 83% of the Indonesian population; the survey included 42,083 individuals aged ≥15 years who had complete data for all study variables (98.2% of the total sample). Utilisation of cardiovascular-related preventive care was analysed for a subsample of 26,612 individuals aged ≥31 years (99.1% of the total subsample); the present study excluded respondents aged ≤31 years because the risk of cardiovascular diseases substantially increase only after age 30 years.

Measurements

The individual's educational level and income were used as indicators of socioeconomic status (SES). Educational level was defined according to the International Standard Classification of Education 2011 issued by UNESCO. Based on the highest level completed by each individual, educational level was categorised into pre-primary, primary, lower secondary, upper secondary and tertiary level.

The level of household consumption was used as a proxy of income. In developing countries, consumption is considered a valid direct measurement of income or household wealth.[22] This measured at household level counted food, non-food consumables, durable goods, spending on education, and housing. These counts were aggregated and transformed into a monthly consumption, which was adjusted for household size to consider the economics of scale.

For peer review only - http://bmjopen.bmj.com/site/about/guidelines.xhtml

We also adjusted for geographical differences in purchasing parity, using Jakarta's poverty line as a reference. Income measurement for different areas was adjusted taking into account variations in the poverty line by province, as well as urban vs. rural place of residence. Data on the poverty line were obtained from the Indonesian Central Bureau of Statistics.

Healthcare utilisation data as collected by the IFLS5 were used; we measured the utilisation of: i) (outpatient) PC, ii) outpatient secondary care, iii) total outpatient care, iv) inpatient care, and v) cardiovascular-related preventive care. PC included any visits to or visits by trained health personnel from a public PC centre, private PC clinic, and/or private PC physician practice. Outpatient secondary care included any visit to a public hospital outpatient care (polyclinics) and private hospital outpatient care. The IFLS5 questionnaire measured all outpatient care that was received during a four-week reference period.

Inpatient care was defined as any use of inpatient care during the previous 12 months for medical purposes, irrespective of the length of hospital stay. This included any use of inpatient care at PC level with inpatient facilities, at public hospitals, or private hospitals. For preventive care utilisation, we focused on cardiovascular diseases-related preventive care because of sizeable contribution of cardiovascular diseases to the overall disease burden in Indonesia.[23] The use of cardiovascular risk factor screening was measured, including blood pressure measurements, cholesterol tests, blood glucose tests, and electrocardiography (ECG) tests during the previous 12 months.

As a proxy of healthcare need, self-assessed health (SAH) was used. SAH is regarded as a health status measurement applicable to different socioeconomic groups. Data on SAH measurement from the IFLS5 survey were used, in which SAH was measured by asking "In general, how is your health?"; the four response categories were "very healthy", "somewhat healthy", "somewhat unhealthy", and "very unhealthy.

Data analysis

For peer review only - http://bmjopen.bmj.com/site/about/guidelines.xhtml

To describe variation in healthcare use among socioeconomic groups, while taking into account differences between these groups in the age and sex structure, we calculated standardised prevalence rate (SPR) for each type of healthcare utilisation by educational levels and income quintiles. SPR was calculated as the number of cases per 100 persons, and was standardised by age and sex the direct method, with the total survey population as the standard population. Next, the rate difference and the rate ratio were calculated based on the SPR of the two lowest SES groups combined, and the two highest SES groups combined, respectively. These SES groups were combined to provide a more stable estimation of the rate difference and the rate ratio between the lower and higher SES groups, respectively. [24] It complements the RII, as the latter takes into account all SES groups separately.

The relative index inequality (RII) was used to estimate the magnitude of inequalities in healthcare utilisation in a more comprehensive way. The RII is a regression-based index that assesses the probability of healthcare use in relationship to the relative hierarchical position of every individual within the socioeconomic hierarchy. A higher RII indicates a stronger association between this hierarchical position and healthcare utilisation, and implies a greater difference in utilisation between higher SES groups compared to lower SES groups. More specifically, RII=1 indicates equality, RII<1 indicates higher utilisation among lower SES, and RII>1 indicates higher utilisation among higher SES.[25] The regression model was adjusted for age, sex and healthcare need, by controlling for SAH in the final model.

To correct for attrition and oversampling, the study sample was weighted with individual weights provided by the IFLS5. We used IBM SPPS Statistics 24 as statistical packacage to analyse the data.

RESULTS

The study sample included slightly more female respondents (51.6%) than males (Table 1). Almost two thirds of the respondents were aged 15-45 years. Males had a generally higher level of education as compared to females. PC was the most frequently used type of healthcare, with 14.6% of the

For peer review only - http://bmjopen.bmj.com/site/about/guidelines.xhtml

respondents reporting that they utilised PC at least once in the previous four weeks. The highest utilisation of preventive care was for blood pressure measurement, with 80.5% of the respondents reporting that their blood pressure was measured during the previous 12 months.

The prevalence rates of PC use were about similar across all educational levels (Table 2, see also online supplementary file Figure 1). Outpatient secondary care utilisation was more frequent among people with a higher educational level compared to those with a lower educational level. For overall inpatient care utilisation, the prevalence rates gradually increased with increasing educational level. A linear association was found between healthcare utilisation and income quintiles for all types of healthcare. This association was particularly strong for utilisation of outpatient secondary care and of inpatient care.

Table 3 (see also online supplementary file Figures 2 and 3) quantifies the magnitude of educational and income inequalities in the utilisation of healthcare. No educational inequalities were found in PC utilisation in the crude analysis, but positive educational inequalities (i.e. higher education associated with higher use rates) emerged after adjusting for SAH (RII 1.13, 95% CI 1.01-1.26). We consistently found positive educational inequalities in all types and levels of health care use after adjusting for SAH. The largest educational inequality was found in outpatient secondary care utilisation (RII 10.35, 95% CI 8.11-13.22).

Positive income inequalities (i.e. higher income associated with higher use rates) were found in all types and levels of healthcare use, especially after adjustment for SAH. Similar to educational inequalities, the largest income inequality was found in outpatient secondary care utilisation (RII 7.43, 95% CI 5.88-9.39). Generally, larger inequalities were found in relationship to income as compared to educational level, except for utilisation of outpatient secondary care.

A consistent linear association was found between prevalence rate of preventive care utilisation and SES (Table 4, and online supplementary file Figure 1). The prevalence rate of blood pressure

For peer review only - http://bmjopen.bmj.com/site/about/guidelines.xhtml

measurement increased incrementally by SES group for both educational level and income quintiles. The prevalence rate of cholesterol tests, blood glucose tests and ECG tests drastically increased from the third highest SES groups to the highest SES groups, both for income and educational level. The differences were larger in relationship to educational level than to income.

Table 5 shows the estimates of the size socioeconomic inequalities in preventive care utilisation (see also online supplementary file Figures 2 and 3). Exceptionally large positive educational inequalities were found in blood glucose tests (RII 30.31, 95% CI 26.13-35.15) and ECG tests (RII 30.90, 95% CI 24.97-38.23). For income inequalities, inequalities in preventive care utilisation were smaller compared to educational inequalities. ECG tests showed the largest income inequality (RII 12.96, 95% CI 10.68-15.73), and blood pressure measurements showed the smallest inequality (RII 3.40, 95% CI 3.04-3.79).

DISCUSSION

This study documented socioeconomic inequalities in healthcare utilisation among the adult population in Indonesia. These inequalities were particularly large for secondary and preventive care. Compared to educational inequalities, income-related inequalities were larger for primary care and inpatient care, but smaller for outpatient secondary and preventive care.

This study was based on a national representative survey with a high response rate (95.3%) and with measurements that matched established international standards.[26] A possible limitation of the study is the measurement of healthcare need, that was limited to SAH. Ideally, we would have used multiple measures of healthcare need, such as self-reported morbidities or health functioning. Although our dataset provided self-reported morbidities and data on health functioning, these are likely to be underestimated in the Indonesian population (particularly in lower SES groups)[27] , and therefore invalid for healthcare need adjustments.

For peer review only - http://bmjopen.bmj.com/site/about/guidelines.xhtml

Because no registry-based data on inequalities in healthcare utilisation in Indonesia are available, we used self-reported use of healthcare. Such healthcare utilisation measures may be subject to recall bias. However, the problem of recall bias might be limited, as the prevalence values of outpatient and inpatient care utilisation from the IFLS5 are close to the national average in Indonesia as reported by the Ministry of Health and data from the National Economic Survey.[28 29]

No other studies have assessed socioeconomic inequalities in general healthcare utilisation in the adult population in Indonesia. The direction and magnitude of inequalities that we observed bear resemblance to the large socioeconomic inequalities in maternal healthcare and child healthcare.[15 19] Our results are also consistent with studies performed in other LMICs showing relatively small inequalities in PC utilisation, and larger inequalities in secondary care.[7-10]

The small socioeconomic inequalities in PC utilisation are probably related to the relatively high supply and geographical distribution of PC providers in Indonesia. Of all registered physicians in Indonesia, 78.4% are general practitioners that mostly practice as public or private providers. In total, there are 9745 public PC centres providing services for the national population with subsidy by local governments.[30 13] Moreover, access to PC is increased by a government-financed NHI program that aims to reduce financial barriers of the poor population to access (primary) healthcare.[29 31] PC is relatively affordable and can be accessed at low cost, even in private practices.[13]

In contrast to PC, the use of secondary care facilities in Indonesia showed considerable inequalities by both educational level and income. For example, individuals with the highest income had 7 times higher odds to use outpatient secondary care compared to those with the lowest income. It is likely that geographical barriers contribute to these inequalities. Because most secondary care facilities and specialists are located in urban areas, the poor need to pay high indirect (e.g. travel and opportunity) costs to access secondary care, even if their medical costs are covered by the NHI program.[29 31] Moreover, there is a limited supply of secondary care specialists; these specialists tend to

For peer review only - http://bmjopen.bmj.com/site/about/guidelines.xhtml

work in private for-profit healthcare providers, which are not contracted by the NHI program. This is likely to result in low financial access for lower SES groups rather then higher SES groups, which may have supplementary private health insurance.[15 18]

We observed inequalities to be larger outpatient secondary care than for inpatient care. A possible explanation is that outpatient secondary care is much more affordable for higher income groups than for lower groups, as the former can pay the service by out-of pocket payment or private health insurance. Lower income groups generally can use outpatient secondary care only by using the government health insurance with its referral system. For inpatient care, however, utilisation costs are significant for higher income groups as well as lower income groups, and usually only affordable via government health insurance and accessible through a referral system.[18]

Inefficient referral procedures could also contribute to inequalities in secondary care utilisation. Even when lower SES groups are entitled to access secondary healthcare, they may lack the knowledge required to obtain a referral, due to the complexity of the administrative procedures in the referral system.[31] Inequalities in secondary care may also be influenced by the preferences and resources that influence the way people utilise healthcare across SES groups.[32] An Indonesian study showed that patients from higher SES groups judged the quality of PC to be low and frequently asked for a referral to secondary care.[33 34]

We observed exceptionally large socioeconomic inequalities in preventive care, particularly by education. For example, individuals who had the highest educational level had 30 times higher odds to have blood glucose test in the previous 12 months compared to those who had the lowest educational level. The individual's level of health literacy may play a major role in their use of preventive care.[35] Those with a relatively low level of health literacy may experience cognitive barriers to make decisions regarding diagnostic tests and treatments that they may need, irrespective of financial, geographic or administrative barriers.[36 37]

For peer review only - http://bmjopen.bmj.com/site/about/guidelines.xhtml

The exceptionally large inequalities in preventive care utilisation may reflect the low priority given to preventive care in Indonesia's health policy. It is currently strongly focused on curative care, resulting in an imbalance in health expenditures on curative and preventive care[38], and the absence of a nationwide preventive care program. The latter is likely to particularly affect those who are least likely to be capable or inclined to use these services, even though they may be in greater need of them.[39 40]

CONCLUSIONS

The findings underline the need to develop comprehensive efforts to tackle significant socioeconomic inequalities in healthcare utilisation in Indonesia. Potential areas of priority include removing financial and geographical barriers by providing the NHI program with universal health coverage, improving the supply and distribution of secondary care services, simplifying the referral system procedure, and developing a nationwide preventive care program. Monitoring healthcare (in)equality will be essential to evaluate the impact of these policies.

Acknowledgement The authors thank the RAND Corporation for providing the dataset of the Indonesian Family Life Survey (IFLS) for this study.

Author Contributions JM conceived the paper. JM and AEK developed the analysis strategy. JM conducted the data analysis. JM, DSK, AEK collectively interpreted the findings. JM prepared the initial draft of the manuscript. JM, DSK, AEK equally contributed to the revision of the manuscript. All authors have read and approved the final manuscript.

Funding This study is funded by the Indonesian Endowment Fund for Education (LPDP), Ministry of Finance, Republic of Indonesia. The funding source has no role in study design, data collection, data analysis and interpretation, manuscript writing, and decision to submit or publish.

Competing Interest None declared

Patient consent Not required

Ethics approval This study is a secondary analysis using the Indonesian Family Life Survey (IFLS) dataset. The IFLS was approved by the Institutional Review Board (IRB) of the Rand Corporation (USA) and the Survey Meter (Indonesia). The data set is publicly available and no personal information can be identified. This study is categorised as being exempt from human research according to the National Institute of Health (NIH).

For peer review only - http://bmjopen.bmj.com/site/about/guidelines.xhtml

Provenance and peer review Not commissioned; externally peer reviewed.

Data sharing statement This study used the Indonesian Family Life Survey (IFLS) dataset provided by RAND Corp. The IFLS dataset is freely accessible at https://www.rand.org/labor/FLS/IFLS.html. Additional unpublished data are available by request to the corresponding author.

Open Access This is an Open Access article distributed in accordance with the Creative Commons Attribution Non Commercial (CC BY-NC 4.0) license, which permits others to distribute, remix, adapt, build upon this work non-commercially, and license their derivative works on different terms, provided the original work is properly cited and the use is non-commercial. See: http://creativecommons.org/licenses/by-nc/4.0/

REFERENCES

1. Oliver A, Mossialos E. Equity of access to health care: outlining the foundations for action. *Journal of epidemiology and community health* 2004;58(8):655-58.
2. Culyer AJ, Wagstaff A. Equity and equality in health and health care. *Journal of health economics* 1993;12(4):431-57.
3. Goddard M, Smith P. Equity of access to health care services: theory and evidence from the UK. *Soc Sci Med* 2001;53 doi: 10.1016/s0277-9536(00)00415-9
4. van Doorslaer E, Masseria C, Koolman X. Inequalities in access to medical care by income in developed countries. *Cmaj* 2006;174 doi: 10.1503/cmaj.050584
5. Walters S, Suhrcke M. Socioeconomic inequalities in health and health care access in central and eastern Europe and the CIS: a review of the recent literature. WHO European Office for Investment for Health and Development2005.
6. Balabanova D, McKee M, Pomerleau J. Health service utilization in the former soviet union: evidence from eight countries. *Health Serv Res* 2004;39 doi: 10.1111/j.1475-6773.2004.00326.x
7. Makinen M, Waters H, Rauch M, et al. Inequalities in health care use and expenditures: empirical data from eight developing countries and countries in transition. *Bulletin of the World Health Organization* 2000;78:55-65.
8. Zhang X, Wu Q, Shao Y, et al. Socioeconomic Inequities in Health Care Utilization in China. *Asia Pacific Journal of Public Health* 2015;27(4):429-38. doi: doi:10.1177/1010539514565446
9. Brinda EM, Attermann J, Gerdtham UG, et al. Socio-economic inequalities in health and health service use among older adults in India: results from the WHO Study on Global AGEing and adult health survey. *Public Health* 2016;141:32-41. doi: 10.1016/j.puhe.2016.08.005 [published Online First: 2016/12/10]
10. Yiengprugsawan V, Carmichael G, Lim LY, et al. Explanation of inequality in utilization of ambulatory care before and after universal health insurance in Thailand. *Health policy and planning* 2011;26(2):105-14. doi: 10.1093/heapol/czq028 [published Online First: 2010/08/26]
11. O'Donnell O. Access to health care in developing countries: breaking down demand side barriers. *Cadernos de saude publica* 2007;23(12):2820-34.
12. Peters DH, Garg A, Bloom G, et al. Poverty and access to health care in developing countries. *Annals of the New York Academy of Sciences* 2008;1136:161-71. doi: 10.1196/annals.1425.011
13. Mahendradhata Y, Trisnantoro L, Listyadewi S, et al. The Republic of Indonesia Health System Review. In: Hort K, Patcharanarumol W, eds. Health Systems in Transition. India: World Health Organization, Regional Office for South-East Asia, 2017.

For peer review only - http://bmjopen.bmj.com/site/about/guidelines.xhtml

14. Pisani E, Olivier Kok M, Nugroho K. Indonesia's road to universal health coverage: a political journey. *Health policy and planning* 2017;32(2):267-76. doi: 10.1093/heapol/czw120
15. Barber SL, Gertler PJ, Harimurti P. Differences In Access To High-Quality Outpatient Care In Indonesia. *Health Affairs* 2007;26(3):w352-w66. doi: 10.1377/hlthaff.26.3.w352
16. Maharani DA, Rahardjo A. Is the utilisation of dental care based on need or socioeconomic status? A study of dental care in Indonesia from 1999 to 2009. *International dental journal* 2012;62(2):90-4. doi: 10.1111/j.1875-595X.2011.00095.x [published Online First: 2012/03/17]
17. Maharani DA. Inequity in dental care utilization in the Indonesian population with a self-assessed need for dental treatment. *The Tohoku journal of experimental medicine* 2009;218(3):229-39. [published Online First: 2009/06/30]
18. Utomo B, Sucahya PK, Utami FR. Priorities and realities: addressing the rich-poor gaps in health status and service access in Indonesia. *International Journal for Equity in Health* 2011;10(1):47. doi: 10.1186/1475-9276-10-47
19. Hosseinpoor AR, Bergen N, Floranita R, et al. State of health inequality: Indonesia. Geneva: World Health Organization, 2017.
20. Indrakesuma T, Janz E, Wai-Po M. A perceived divide : how Indonesians perceive inequality and what they want done about it. Jakarta: The World Bank 2015.
21. Strauss J, Witoelar F, Sikoki B. The Fifth Wave of the Indonesia Family Life Survey: Overview and Field Report: RAND Labor and Population, 2016.
22. O'Donnel O, Doorslaer Ev, Wagstaff A, et al. Analyzing Health Equity Using Household Survey Data: A Guide to Techniques and Their Implementation. Washington: The World Bank Institute 2008.
23. Riley LM, Cowan M. Noncommunicable Diseases Country Profile 2014. Geneva: World Health Organization, 2014.
24. Hosseinpoor AR, Bergen N. Handbook on Health Inequality Monitoring with a special focus in low-middle income countries Geneva: Worrld Health Organization, 2013.
25. Mackenbach JP, Kunst AE. Measuring the magnitude of socio-economic inequalities in health: an overview of available measures illustrated with two examples from Europe. *Soc Sci Med* 1997;44(6):757-71.
26. Galobardes B, Lynch J, Smith GD. Measuring socioeconomic position in health research. *British Medical Bulletin* 2007;81-82(1):21-37. doi: 10.1093/bmb/ldm001
27. Vellakkal S, Millett C, Basu S, et al. Are estimates of socioeconomic inequalities in chronic disease artefactually narrowed by self-reported measures of prevalence in low-income and middle-income countries? Findings from the WHO-SAGE survey. *Journal of epidemiology and community health* 2014:jech-2014-204621.
28. Riset Kesehatan Dasar 2013. Jakarta: Badan Penelitian dan Pengembangan Kementerian Kesehatan Republik Indonesia 2013.
29. Sparrow R, Suryahadi A, Widyanti W. Social health insurance for the poor: Targeting and impact of Indonesia's Askeskin programme. *Social Science & Medicine* 2013;96:264-71. doi: http://doi.org/10.1016/j.socscimed.2012.09.043
30. Profil Kesehatan Indonesia 2015. Jakarta: Kementerian Kesehatan Republik Indonesia, 2016.
31. Harimurti P, Pambudi E, Pigazzini A, et al. The Nuts & Bolts of Jamkesmas, Indonesia's Government-financed Health Coverage Program for the Poor and Near-Poor. UNICO Studies Series. Washington DC: The World Bank, 2013.
32. Levesque J-F, Harris MF, Russell G. Patient-centred access to health care: conceptualising access at the interface of health systems and populations. *International Journal for Equity in Health* 2013;12:18-18. doi: 10.1186/1475-9276-12-18

For peer review only - http://bmjopen.bmj.com/site/about/guidelines.xhtml

33. Istiono W, Claramita M, Ekawati FM, et al. Physician's self-perceived abilities at primary care settings in Indonesia. *Journal of Family Medicine and Primary Care* 2015;4(4):551-58. doi: 10.4103/2249-4863.174286
34. Sari MI, Prabandari YS, Claramita M. Physicians’ professionalism at primary care facilities from patients’ perspective: The importance of doctors’ communication skills. *Journal of Family Medicine and Primary Care* 2016;5(1):56-60. doi: 10.4103/2249-4863.184624
35. Gottfredson LS. Intelligence: is it the epidemiologists' elusive" fundamental cause" of social class inequalities in health? *Journal of personality and social psychology* 2004;86(1):174.
36. Chen JZ, Hsu HC, Tung HJ, et al. Effects of health literacy to self-efficacy and preventive care utilization among older adults. *Geriatrics & gerontology international* 2013;13(1):70-6. doi: 10.1111/j.1447-0594.2012.00862.x [published Online First: 2012/06/08]
37. Huang C, Liu CJ, Pan XF, et al. Correlates of unequal access to preventive care in China: a multilevel analysis of national data from the 2011 China Health and Nutrition Survey. *BMC Health Serv Res* 2016;16:177. doi: 10.1186/s12913-016-1426-2 [published Online First: 2016/05/14]
38. Widjaja FF. Universal health coverage in Indonesia–the forgotten prevention. *Med J Indones* 2014;23(3)
39. Rodin D, Stirbu I, Ekholm O, et al. Educational inequalities in blood pressure and cholesterol screening in nine European countries. *J Epidemiol Community Health* 2012;66(11):1050-5. doi: 10.1136/jech-2011-200273 [published Online First: 2012/01/17]
40. Palència L, Espelt A, Rodríguez-Sanz M, et al. Socio-economic inequalities in breast and cervical cancer screening practices in Europe: influence of the type of screening program. *International Journal of Epidemiology* 2010;39(3):757-65. doi: 10.1093/ije/dyq003

For peer review only

Table 1. Basic characteristics of the study population.

Variables	Total		Male		Female	
	n	%	n	%	n	%
**Gender**						
Male	20374	48.4	-	-	-	-
Female	21709	51.6	-	-	-	-
**Age group (in years)**						
15-30	12471	29.6	6436	31.6	6035	27.8
31-45	14049	33.4	6545	32.1	7503	34.6
46-60	10280	24.4	4973	24.4	5306	24.4
>60	5283	12.6	2419	11.9	2864	13.2
**Education level**						
Pre-primary	9868	23.4	3977	19.5	5891	27.1
Primary	9993	23.7	4855	23.8	5138	23.7
Lower secondary	8082	19.2	4041	19.8	4041	18.6
Upper secondary	10731	25.2	5894	28.9	4838	22.3
Tertiary	3409	8.1	1607	7.9	1802	8.3
**Income**[a]						
1st quintile (230-1300)	8417	20.0	4050	19.9	4367	20.1
2nd quintile (1300-1830)	8418	20.0	3997	19.6	4421	20.4
3rd quintile (1830-2520)	8415	20.0	4056	19.9	4359	20.1
4th quintile (2520-3830)	8417	20.0	4159	20.4	4258	19.6
5th quintile (3830-55400)	8416	20.0	4111	20.2	4305	19.8
**Self-assessed health**						
Very healthy	8137	19.3	4362	21.4	3775	17.4
Somewhat healthy	24757	58.8	12179	59.8	12578	57.9
Somewhat unhealthy	8447	20.1	3513	17.2	4934	22.7
Very unhealthy	742	1.8	320	1.6	422	1.9
**Outpatient care utilisation**						
Primary care	6155	14.6	2006	9.8	4149	19.1
Secondary care	1022	2.4	427	2.1	595	2.7
Total	6864	16.3	2323	11.4	4541	20.9
**Inpatient care utilisation**						
Overall	1937	4.6	591	2.9	1346	6.2
**Preventive care utilisation (age ≥ 31 years)**						
Blood pressure screening	21663	80.5	9254	74.3	12409	85.4
Cholesterol screening	4678	17.4	1951	15.7	2727	18.9
Blood glucose screening	4142	15.4	1855	14.9	2287	15.8
Electrocardiography test	1723	6.4	876	7.0	847	5.9

[a]Income in thousands Indonesian Rupiah (IDR)

For peer review only - http://bmjopen.bmj.com/site/about/guidelines.xhtml

Table 2. Standardised prevalence rate (SPR) of healthcare utilisation by socioeconomic status.

	Outpatient care (SPR, 95% CI)†			Inpatient care (SPR, 95% CI)
	Primary	Secondary	Total	Overall
**Education**				
Pre-primary	14.47(13.78-15.18)	1.31(1.12-1.53)	15.22(14.52-15.95)	3.07(2.77-3.40)
Primary	14.93(14.19-15.70)	2.03(1.76-2.32)	16.07(15.30-16.87)	4.60(4.18-5.04)
Lower secondary	15.00(14.12-15.91)	1.88(1.57-2.22)	16.38(15.47-17.33)	4.31(3.86-4.81)
Upper secondary	14.38(13.64-15.16)	3.39(3.03-3.79)	16.99(16.18-17.84)	6.12(5.63-6.64)
Tertiary	14.37(13.12-15.71)	6.26(5.44-7.17)	18.97(17.53-20.50)	6.93(6.04-7.91)
**Income**				
1st quintile	11.71(11.02-12.42)	1.32(1.09-1.57)	12.54(11.83-13.29)	2.99(2.65-3.35)
2nd quintile	14.11(13.34-14.92)	1.44(1.20-1.71)	14.73(13.94-15.55)	3.76(3.37-4.18)
3rd quintile	15.36(14.54-16.22)	2.00(1.71-2.33)	16.75(15.89-17.64)	4.24(3.81-4.70)
4th quintile	15.73(14.88-16.62)	2.91(2.55-3.31)	18.57(17.64-19.53)	5.13(4.64-5.65)
5th quintile	16.13(15.24-17.06)	4.97(4.48-5.50)	19.83(18.74-20.85)	7.56(6.94-8.21)

†Prevalence rate per 100 persons, age and sex standardised to the total population.

Table 3. Socioeconomic inequalities in the utilisation of various types and levels of healthcare.

	Type of care	Level of care	SPR (95% CI)†		Rate difference	Rate ratio	RII (95% CI), adjusted for age, sex	RII (95% CI), adjusted for age, sex, SAH
			Two lowest groups	Two highest groups				
Education	Outpatient	Primary	14.68(14.18-15.20)	14.38(13.74-15.05)	-0.30	0.98	0.99 (0.98-1.01)	1.13 (1.01-1.26)
		Secondary	1.64(1.47-1.82)	2.88 (2.59-3.19)	1.24	1.76	7.89 (6.33-9.85)	10.35 (8.11-13.22)
		Total	15.62(15.09-16.15)	17.51(16.80-18.25)	1.89	1.12	1.35 (1.24-1.46)	1.59 (1.44-1.77)
	Inpatient	Overall	3.74(3.48-4.01)	6.31(5.88-6.76)	2.57	1.69	2.38 (1.97-2.76)	2.78 (2.32-3.32)
Income	Outpatient	Primary	12.88(12.35-13.42)	16.25(15.62-16.89)	3.37	1.26	1.50 (1.39-1.62)	1.68 (1.52-1.85)
		Secondary	1.36(1.20-1.54)	4.69(4.35-5.04)	3.33	3.45	6.61 (5.29-8.25)	7.43 (5.88-9.39)
		Total	13.61(13.08-14.16)	19.18(18.50-19.88)	5.57	1.41	1.80 (1.67-1.94)	2.15 (1.96-2.36)
	Inpatient	Overall	3.36(3.10-3.63)	6.30(5.91-6.71)	2.94	1.88	2.94 (2.52-3.43)	3.11 (2.63-3.66)

†Prevalence rate per 100 persons, age and sex standardised to the total population.

For peer review only

Table 4. Standardised prevalence rate (SPR) of preventive care utilisation by socioeconomic status.

	Preventive care activity (SPR, 95% CI)†			
	Blood pressure	Cholesterol	Blood glucose	ECG
**Education**				
Pre-primary	72.40(70.54-74.30)	8.85(8.24-9.49)	6.85(6.32-7.41)	2.51(2.19-2.86)
Primary	79.54(77.47-81.65)	10.78(10.03-11.58)	10.59(9.84-11.39)	4.00(3.54-4.50)
Lower secondary	82.72(79.88-85.63)	18.03(16.67-19.48)	15.44(14.17-16.79)	4.95(4.25-5.73)
Upper secondary	86.66(84.27-89.10)	26.83(25.44-28.27)	24.72(23.39-26.10)	10.80(9.94-11.71)
Tertiary	92.33(88.45-96.35)	44.72(41.97-47.60)	43.38(40.66-46.23)	20.99(19.13-22.99)
**Income**				
1st quintile	73.55(71.27-75.88)	7.83(7.11-8.60)	6.12(5.49-6.81)	2.71(2.29-3.18)
2nd quintile	76.77(74.45-79.15)	12.12(11.21-13.08)	10.14(9.30-11.02)	3.37(2.90-3.90)
3rd quintile	80.60(78.22-83.03)	14.37(13.37-15.42)	12.63(11.70-13.63)	4.64(4.08-5.26)
4th quintile	83.68(81.25-86.16)	20.34(19.14-21.59)	18.41(17.27-19.60)	7.13(6.43-7.89)
5th quintile	87.87(85.38-90.41)	32.69(31.17-34.26)	30.09(28.64-31.60)	14.43(13.43-15.49)

†Prevalence rate per 100 persons, age and sex standardised to the total population

Table 5. Socioeconomic inequalities in the use of various preventive care activities.

	Activity	SPR (95% CI)†		Rate difference	Rate ratio	RII (95% CI) adjusted for age, sex	RII (95% CI) adjusted for age, sex, SAH
		Two lowest groups	Two highest groups				
**Education**	Blood pressure	75.77(74.38-77.17)	88.28(86.24-90.36)	12.51	1.17	6.37 (5.61-7.24)	6.67(5.87-7.59)
	Cholesterol	9.69(9.22-10.19)	32.12(30.85-33.44)	22.43	3.31	18.17(15.91-20.74)	21.27(18.53-24.41)
	Blood glucose	8.47(8.03-8.94)	30.21(28.98-31.49)	21.74	3.57	24.61(21.36-28.36)	30.31(26.13-35.15)
	ECG	3.17(2.90-3.46)	13.74(12.92-14.60)	10.57	4.33	25.45(20.72-31.20)	30.90(24.97-38.23)
**Income**	Blood pressure	75.17(73.54-76.83)	86.28(84.54-88.05)	11.11	1.15	3.34(2.99-3.72)	3.40(3.04-3.79)
	Cholesterol	8.61(8.06-9.17)	26.54(25.57-25.53)	17.93	3.08	9.20(8.15-10.40)	9.76 (8.63-11.02)
	Blood glucose	8.11(7.59-8.66)	24.29(23.36-35.54)	16.18	3.00	10.81(9.49-12.30)	11.59(10.18-13.20)
	ECG	3.03(2.71-3.37)	10.78(10.14-11.40)	7.73	3.55	12.42(10.23-15.07)	12.96(10.68-15.73)

†Prevalence rate per 100 persons, age and sex standardised to the total population

For peer review only - http://bmjopen.bmj.com/site/about/guidelines.xhtml

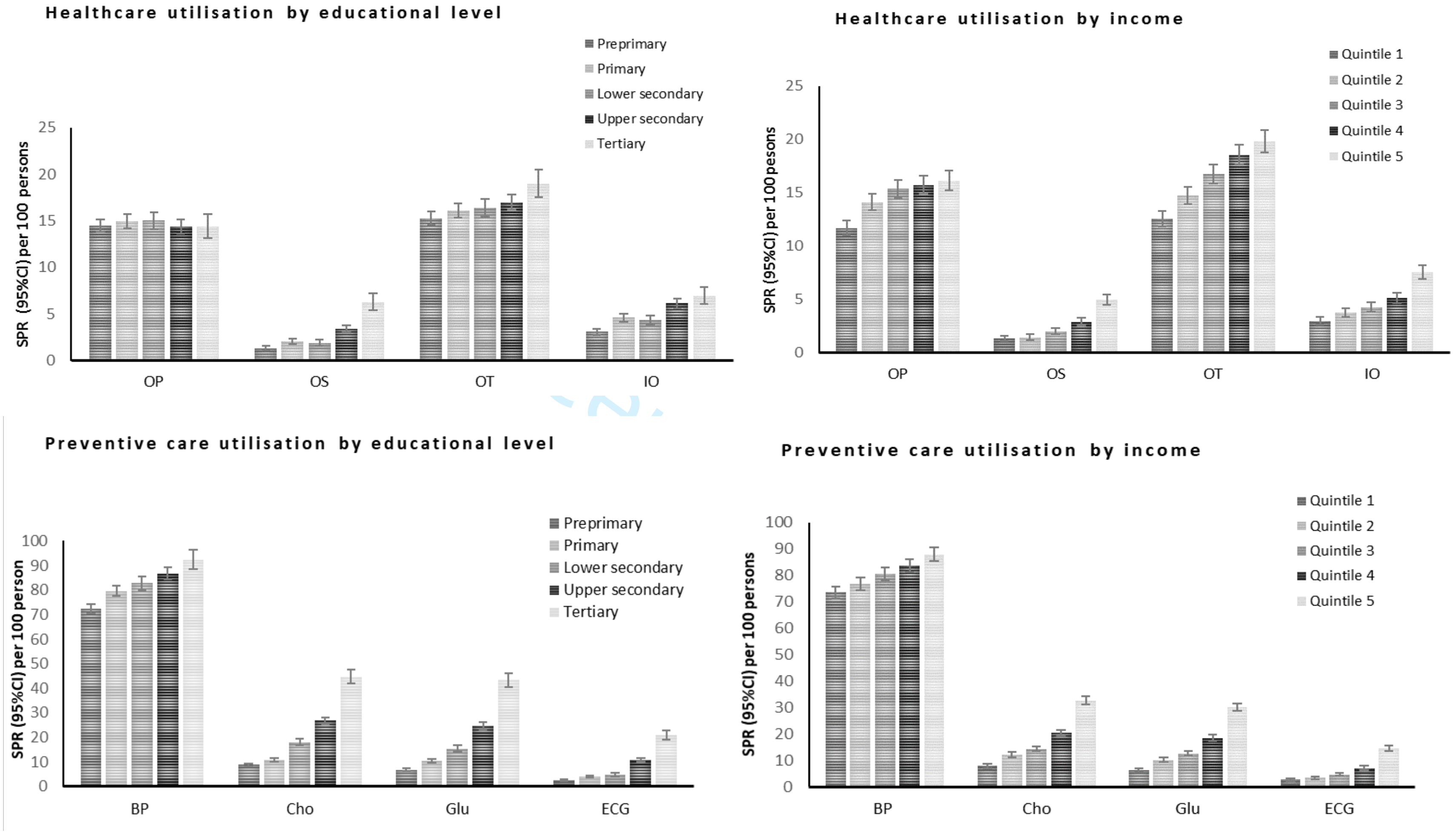

Figure 1. Standardised prevalence rate (95%CI) for healthcare and preventive care utilisation. Prevalence rate is per 100 persons, standardised by age and sex to total population. OP: Outpatient primary care; OS: Outpatient secondary care; OT: Outpatient total; IO: Inpatient overall; BP: Blood pressure; Cho: Cholesterol; Glu: Blood glucose; ECG: Electrocardiograph.

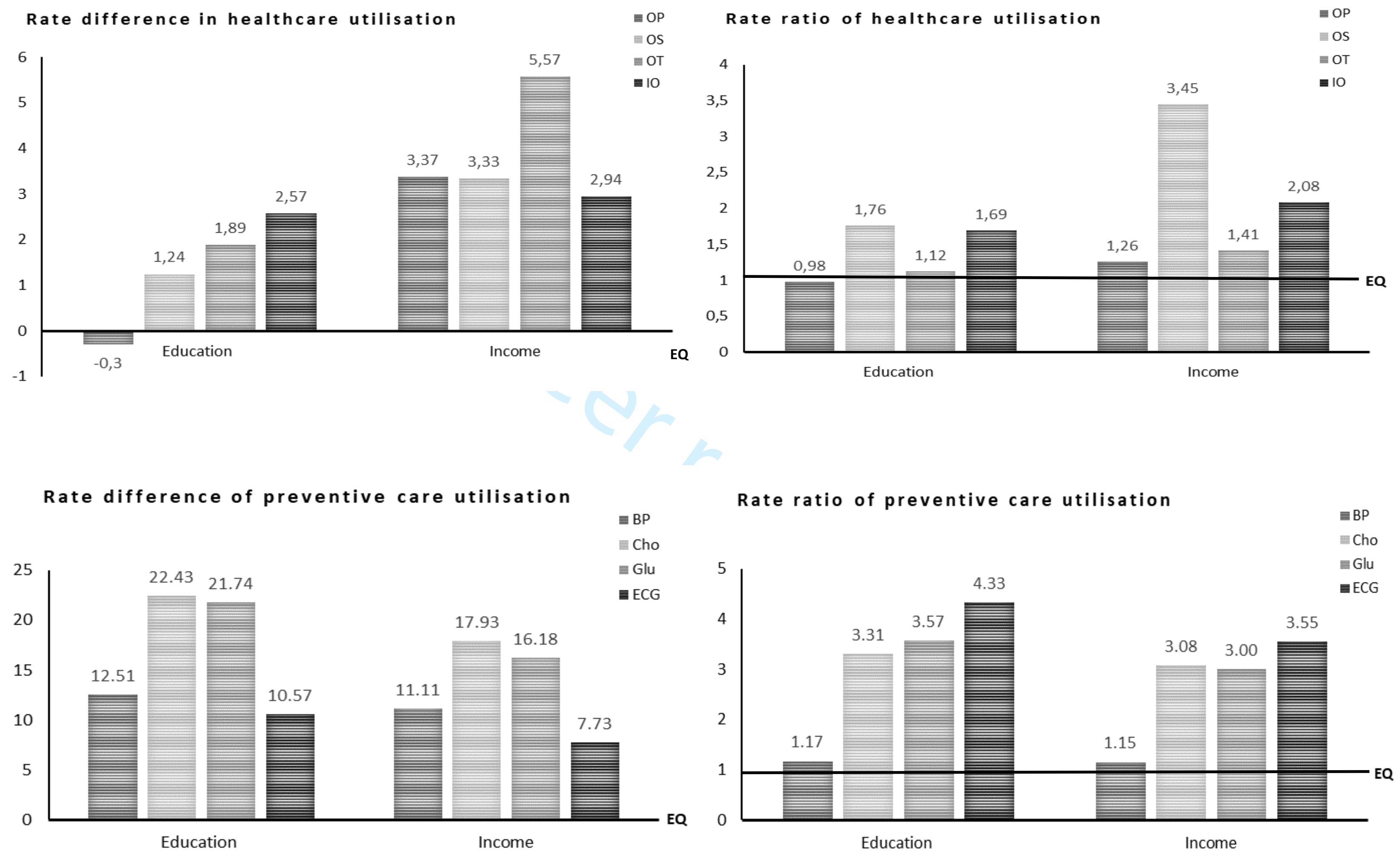

Figure 2. **Simple measurement of absolute (rate difference) and relative (rate ratio) inequalities in healthcare and preventive care utilisation between two highest and two lowest groups of SES. Rate difference is per 100 persons. OP: Outpatient primary care; OS: Outpatient secondary care; OT: Outpatient total; IO: Inpatient overall; BP: Blood pressure; Cho: Cholesterol; Glu: Blood glucose; ECG: Electrocardiograph; EQ: Equality line.**

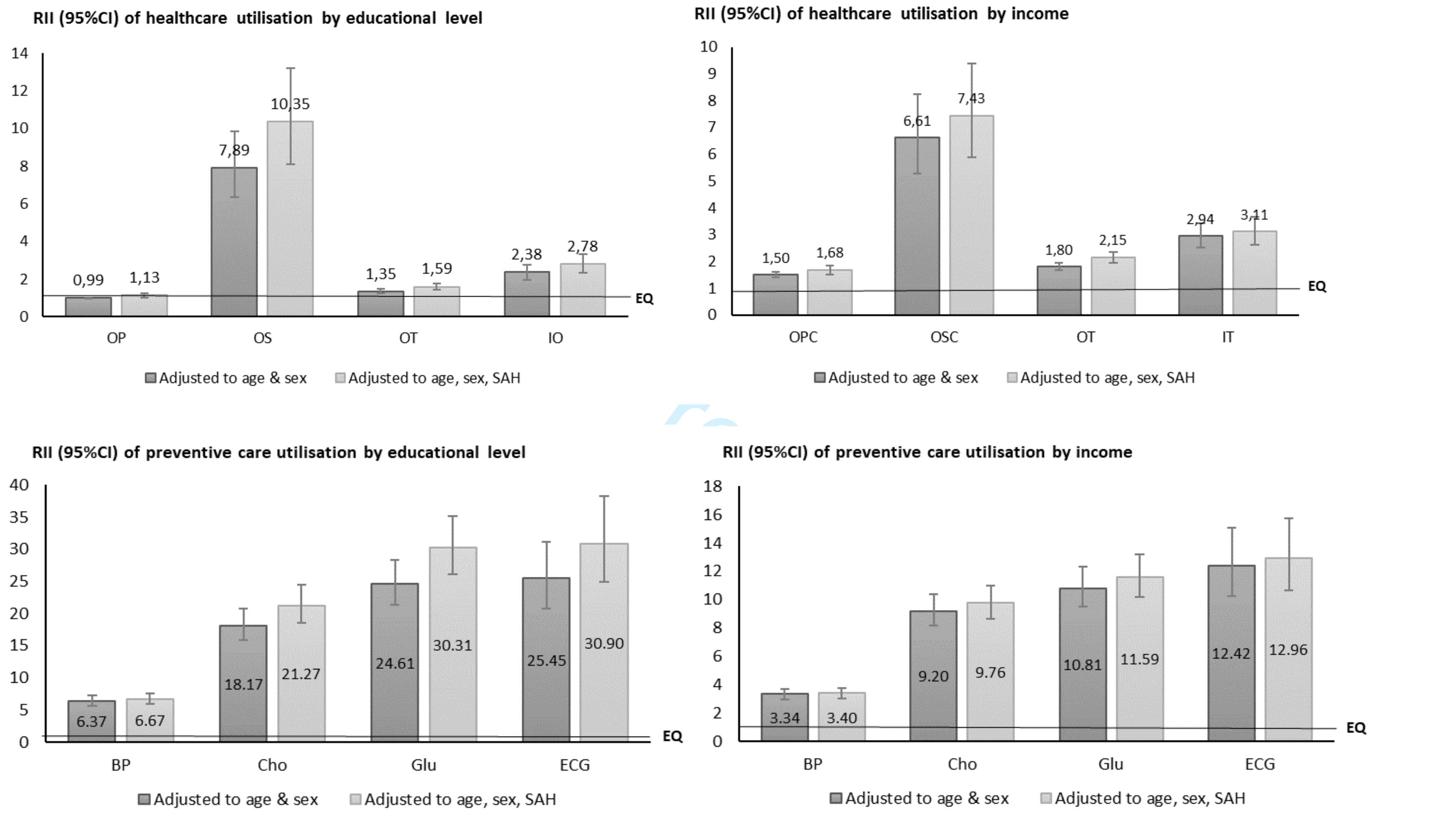

Figure 3. Relative index inequality (95%CI) of healthcare and preventive care utilisation by educational level and income. OP: Outpatient primary care; OS: Outpatient secondary care; OT: Outpatient total; IO: Inpatient overall; BP: Blood pressure; Cho: Cholesterol; Glu: Blood glucose; ECG: Electrocardiograph; EQ: Equality line; SAH: Self-assessed health.

STROBE Statement—Checklist of items that should be included in reports of ***cross-sectional studies***

	Item No	Recommendation
**Title and abstract**	1	(*a*) Indicate the study's design with a commonly used term in the title or the abstract **[see methods section of abstract page 2]**
		(*b*) Provide in the abstract an informative and balanced summary of what was done and what was found **[see methods and results section of abstract page 2]**
**Introduction**		
Background/rationale	2	Explain the scientific background and rationale for the investigation being reported **[page 4]**
Objectives	3	State specific objectives, including any pre-specified hypotheses [**page 5]**
**Methods**		
Study design	4	Present key elements of study design early in the paper **[page 6]**
Setting	5	Describe the setting, locations, and relevant dates, including periods of recruitment, exposure, follow-up, and data collection **[page 6]**
Participants	6	(*a*) Give the eligibility criteria, and the sources and methods of selection of participants **[page 6]**
Variables	7	Clearly define all outcomes, exposures, predictors, potential confounders, and effect modifiers. Give diagnostic criteria, if applicable **[page 7]**
Data sources/ measurement	8*	For each variable of interest, give sources of data and details of methods of assessment (measurement). Describe comparability of assessment methods if there is more than one group **[page 7]**
Bias	9	Describe any efforts to address potential sources of bias **[page 6]**
Study size	10	Explain how the study size was arrived at **[page 6]**
Quantitative variables	11	Explain how quantitative variables were handled in the analyses. If applicable, describe which groupings were chosen and why **[page 7]**
Statistical methods	12	(*a*) Describe all statistical methods, including those used to control for confounding **[page 8]**
		(*b*) Describe any methods used to examine subgroups and interactions **[N/A]**
		(*c*) Explain how missing data were addressed **[page 6]**
		(*d*) If applicable, describe analytical methods taking account of sampling strategy **[page 8]**
		(*e*) Describe any sensitivity analyses **[N/A]**
**Results**		
Participants	13*	(a) Report numbers of individuals at each stage of study—eg numbers potentially eligible, examined for eligibility, confirmed eligible, included in the study, completing follow-up, and analysed **[N/A]**
		(b) Give reasons for non-participation at each stage **[N/A]**
		(c) Consider use of a flow diagram **[N/A]**
Descriptive data	14*	(a) Give characteristics of study participants (eg demographic, clinical, social) and information on exposures and potential confounders [**page 8, table 1 (page 17]**
		(b) Indicate number of participants with missing data for each variable of interest **[N/A]**
Outcome data	15*	Report numbers of outcome events or summary measures **[page 8-9, table 2,4 (page 18,19]**
Main results	16	(*a*) Give unadjusted estimates and, if applicable, confounder-adjusted estimates and their precision (eg, 95% confidence interval). Make clear which confounders were

For peer review only

		adjusted for and why they were included **[page 8-10, table 3,5 (page 18,19)]**
		(*b*) Report category boundaries when continuous variables were categorized **[N/A]**
		(*c*) If relevant, consider translating estimates of relative risk into absolute risk for a meaningful time period **[N/A]**
Other analyses	17	Report other analyses done—eg analyses of subgroups and interactions, and sensitivity analyses **[N/A]**
**Discussion**		
Key results	18	Summarise key results with reference to study objectives **[page 10]**
Limitations	19	Discuss limitations of the study, taking into account sources of potential bias or imprecision. Discuss both direction and magnitude of any potential bias **[page 10-11]**
Interpretation	20	Give a cautious overall interpretation of results considering objectives, limitations, multiplicity of analyses, results from similar studies, and other relevant evidence **[page 12-13]**
Generalisability	21	Discuss the generalisability (external validity) of the study results **[page 13]**
**Other information**		
Funding	22	Give the source of funding and the role of the funders for the present study and, if applicable, for the original study on which the present article is based **[declaration section, page 13]**

*Give information separately for exposed and unexposed groups.

Note: An Explanation and Elaboration article discusses each checklist item and gives methodological background and published examples of transparent reporting. The STROBE checklist is best used in conjunction with this article (freely available on the Web sites of PLoS Medicine at http://www.plosmedicine.org/, Annals of Internal Medicine at http://www.annals.org/, and Epidemiology at http://www.epidem.com/). Information on the STROBE Initiative is available at www.strobe-statement.org.

For peer review only

For peer review only - http://bmjopen.bmj.com/site/about/guidelines.xhtml

BMJ Open

Socioeconomic inequalities in healthcare utilisation in Indonesia: a comprehensive survey-based overview

Journal:	*BMJ Open*
Manuscript ID	bmjopen-2018-026164.R1
Article Type:	Research
Date Submitted by the Author:	08-Mar-2019
Complete List of Authors:	Mulyanto, Joko; Amsterdam UMC, University of Amsterdam, Amsterdam Public Health research institute, Public Health; Faculty of Medicine Universitas Jenderal Soedirman, Public Health and Community Medicine<br>Kringos, Dionne Sofia; Amsterdam UMC, University of Amsterdam, Amsterdam Public Health research institute, Public Health<br>Kunst, Anton; Amsterdam UMC, University of Amsterdam, Amsterdam Public Health research institute, Public Health
**Primary Subject Heading**:	Health services research
Secondary Subject Heading:	Health policy
Keywords:	Socioeconomic, Inequalities, Access, Healthcare, Indonesia

SCHOLARONE™
Manuscripts

Socioeconomic inequalities in healthcare utilisation in Indonesia: a comprehensive survey-based overview

Joko Mulyanto[1,2], Dionne S Kringos[1], Anton E Kunst[1]

[1]Department of Public Health, Amsterdam UMC, University of Amsterdam, Amsterdam Public Health research institute, Amsterdam, the Netherlands.
[2]Department of Public Health and Community Medicine, Faculty of Medicine, Universitas Jenderal Soedirman, Purwokerto, Indonesia

Corresponding author
Joko Mulyanto, Department of Public Health, Amsterdam UMC, University of Amsterdam, PO Box 22660 1100 DD, Amsterdam, the Netherlands; Telephone: +31-20-5664642; j.mulyanto@amc.uva.nl

For peer review only

ABSTRACT

Objective Monitoring inequality in healthcare utilisation is essential to reduce persistent inequalities in health in lower-middle income countries. This study aimed to assess socioeconomic inequalities in the utilisation of primary care, secondary care, and preventive care in Indonesia.

Methods A cross-sectional study was conducted using data from the 2014 Indonesia Family Life Survey with a total of 42,083 adult participants. Socioeconomic status (SES) was measured by educational level and income. Healthcare utilisation was measured in: i) primary care, ii) outpatient in secondary care, iii) inpatient care, and iii) cardiovascular-related preventive care. The magnitude of inequalities was measured using the relative index of inequality (RII).

Results Small educational inequalities were found for primary care utilisation (RII 1.13, 95% CI 1.01-1.26). Larger educational inequalities were found for outpatient secondary care (RII 10.35, 95% CI 8.11-13.22) and inpatient care (RII 2.78, 95% CI 2.32-3.32). The largest educational inequalities were found for preventive care, particularly regarding blood glucose tests (RII 30.31, 95% CI 26.13-35.15) and electrocardiography tests (RII 30.90, 95% CI 24.97-38.23). Compared to educational inequalities, income inequalities were larger for primary care (RII 1.68, 95% CI 1.52-1.85) and inpatient care (RII 3.11, 95% CI 2.63-3.66), but not for outpatient secondary care and preventive care.

Conclusions Socioeconomic inequalities in healthcare utilisation in Indonesia are particularly large in secondary and preventive care. Therefore, it is recommended to prioritise policies focused on improving timely, geographical and financial access to secondary and preventive care for lower SES groups.

Keywords socioeconomic: inequalities; access; healthcare: Indonesia

For peer review only

For peer review only - http://bmjopen.bmj.com/site/about/guidelines.xhtml

ARTICLE SUMMARY

Strengths and limitations of this study

- This study was based on a nationally representative survey with a high response rate and with measurements that matched established international standards.
- Few studies have investigated inequalities in healthcare utilisation in Indonesia
- The measurement of healthcare need was limited to self-assessed health.
- The measurement of healthcare utilisation was based on self-reported data which might be subject to recall bias.

For peer review only

For peer review only - http://bmjopen.bmj.com/site/about/guidelines.xhtml

INTRODUCTION

Equal use of healthcare for equal need is essential to improve population health and is, therefore, an objective for most healthcare systems. Monitoring inequality in healthcare utilisation is essential to assess the performance of a healthcare system, and ultimately, to reduce persistent inequalities in health.[1] To monitor inequality in use in these terms, healthcare utilisation should be adjusted for self-assessed health or morbidities, as determinants of healthcare need.[2 3]

There is evidence of inequalities in healthcare utilisation in developed countries, despite universal healthcare coverage.[4] For example, both in Western and Eastern Europe, inequalities in healthcare utilisation exist for certain types of healthcare. In Eastern Europe, the rapid transition of the healthcare system since the late 1990s after the fall of Communism may have been conducive to large inequalities.[5 6]

Lower-middle income countries (LMICs) also experience inequalities in healthcare utilisation, and especially in secondary care, as shown by an international comparative study in Asia, Africa and Latin America[7]; and studies in China and India.[8 9] Significant inequalities in healthcare utilisation are also found in Thailand, despite universal healthcare coverage since 2005.[10] For several reasons, sizeable inequalities in healthcare utilisation may also exist in other LMICs. Many of LMICs are struggling to provide universal healthcare coverage, resulting in persisting financial barriers to access healthcare. Furthermore, inadequate supply and unequal geographic distribution of healthcare facilities cause greater barriers to the use of these facilities by people living in remote places and with limited resources. Moreover, large inequalities in the quality of healthcare that is received may result from poor stewardship, low financial investments in the healthcare system, and suboptimal quality of a broad range of healthcare services.[11 12]

Indonesia is an LMIC with a population of 262 million people that are distributed across ≥17,000 islands, and with diverse ethnic and religious backgrounds. Indonesia's healthcare system is a mixture of public and private healthcare delivery systems. The size and role of the private-commercial healthcare

For peer review only - http://bmjopen.bmj.com/site/about/guidelines.xhtml

market have increased during the last decade. Total health expenditure in 2014 was 2.8% of the gross domestic product, of which 47% originated from out-of-pocket payments. Since 1999, the government has provided health insurance for the poor, and in 2014 introduced the National Health Insurance (NHI) program to remove financial barriers to access basic healthcare services for the entire population by 2019.[13]

Current policy to achieve equal access in healthcare in Indonesia is focusing on the expansion of the NHI program.[14] However, over the years, progress towards universal health coverage has been uneven and iterative and consistently driven by domestic political interests as opposed to technical considerations.[15] The dominance of political interest is also reflected in the government evaluation of the NHI program which emphasised the overall coverage (NHI membership) of the population and paid less attention to the issue of the actual access distribution such as inequality among various population groups.[16]

In terms of preventive care, communicable diseases are still the government's priority with the improvement of universal child immunisation as the main focus.[16] Until recently, Indonesia did not implement a systematic policy or programs for the prevention of cardiovascular diseases or other main non-communicable diseases (NCDs). [13] Furthermore, the NHI program put much emphasis on curative care, which makes the utilisation of preventive care likely depend more on personal resources than on collective efforts.[17]

Lack of information which comprehensively assess the current situation of inequalities in healthcare utilisation in Indonesia may contribute to the low attention of the government in this issue. During the last decade, only a few studies have investigated inequalities in healthcare utilisation in Indonesia. Previous studies focused on the inequalities in maternal and child-related healthcare and dental care.[18-21] A recent report from the WHO stated that large inequalities in maternal and child healthcare persist in Indonesia, in addition to geographic inequalities in the healthcare infrastructure,

For peer review only - http://bmjopen.bmj.com/site/about/guidelines.xhtml

particularly between the different provinces.[22] A recent study showed wealth-related inequalities in Indonesia in the use of healthcare, particularly in secondary care. However, this study did not assess inequalities in relation to other SES indicators such as educational level, nor did it consider inequalities in preventive care utilisation.[23]

No studies have empirically assessed socioeconomic inequalities (in terms of both educational level and income) in general healthcare utilisation in Indonesia particularly for preventive care utilisation. The present study aimed to fill in this gap of evidence. Using a large-scale national interview survey, we aimed to provide a comprehensive overview of socioeconomic inequalities in the utilisation of primary care, secondary care and preventive care in Indonesia. Findings from this study would be particularly beneficial for policymakers to assess the progress of the current efforts to reduce inequalities and also for policy development to further address inequalities in healthcare utilisation in Indonesia.

METHODS

Study design and data sources

Data was used from the fifth wave of the Indonesia Family Life Survey (IFLS5) which was conducted in 2014 by the RAND Corporation (USA). The data and supporting documents such as the survey protocol and questionnaires are publicly accessible through RAND's website.[24] The IFLS5 was approved by the relevant ethical review committees in the USA and Indonesia. It collected data from 13 Indonesian provinces that together represent 83% of the Indonesian population; the survey included 42,083 individuals aged 15 years or older who had complete data for all study variables (98.2% of the total sample). For the analysis of cardiovascular-related preventive care utilisation, we included 26,612 individuals aged 31 years or older, which is 89.9% of the total number of individuals aged 31 years or older in the sample (29,612 individuals) and 63.2% of the total all-age sample (42,083 individuals). The

For peer review only - http://bmjopen.bmj.com/site/about/guidelines.xhtml

present study excluded respondents aged 31 years or older because the risk of cardiovascular diseases substantially increases only after the age of 30 years.

Measurements

The individual's educational level and income were used as indicators of socioeconomic status (SES). Educational level was defined according to the International Standard Classification of Education 2011 issued by UNESCO. Based on the highest level completed by each individual, educational level was categorised into pre-primary, primary, lower secondary, upper secondary and tertiary level.

The level of household consumption was used as a proxy of income. In developing countries, consumption is considered a valid direct measurement of income or household wealth.[25] This measured at household level counted food, non-food consumables, durable goods, spending on education, and housing. These counts were aggregated and transformed into a monthly consumption, which was adjusted for household size to consider the economics of scale.

We also adjusted for geographical differences in purchasing parity, using Jakarta's poverty line as a reference. Income measurement for different areas was adjusted taking into account variations in the poverty line by province, as well as urban vs. rural place of residence. Data on the poverty line were obtained from the Indonesian Central Bureau of Statistics.

Healthcare utilisation data as collected by the IFLS5 were used; we measured the utilisation of: i) (outpatient) primary care, ii) outpatient secondary care, iii) total outpatient care, iv) inpatient care, and v) cardiovascular-related preventive care. Primary care included any visits to or visits by trained health personnel from a public primary care centre, private primary care clinic, and/or private primary care physician practice. Outpatient secondary care included any visit to a public hospital outpatient care (polyclinics) and private hospital outpatient care. The IFLS5 questionnaire measured all outpatient care that was received during a four-week reference period.

For peer review only - http://bmjopen.bmj.com/site/about/guidelines.xhtml

Inpatient care was defined as any use of inpatient care during the previous 12 months for medical purposes, irrespective of the length of hospital stay. This included any use of inpatient care at primary care level with inpatient facilities, at public hospitals, or private hospitals. For preventive care utilisation, we focused on cardiovascular diseases-related preventive care because of the sizeable contribution of cardiovascular diseases to the overall disease burden in Indonesia.[26] The use of cardiovascular risk factor screening was measured, including blood pressure measurements, cholesterol tests, blood glucose tests, and electrocardiography (ECG) tests during the previous 12 months.

As a proxy of healthcare need, self-assessed health (SAH) was used. SAH is regarded as a health status measurement applicable to different socioeconomic groups. Data on SAH measurement from the IFLS5 survey were used, in which SAH was measured by asking “In general, how is your health?”; the four response categories were “very healthy”, “somewhat healthy”, “somewhat unhealthy”, and “very unhealthy.

Data analysis

To describe variation in healthcare use among socioeconomic groups, while taking into account differences between these groups in the age and sex structure, we calculated standardised prevalence rate (SPR) for each type of healthcare utilisation by educational levels and income quintiles. SPR was calculated as the number of cases per 100 persons and was standardised by age and sex the direct method, with the total survey population as the standard population. Next, the rate difference and the rate ratio were calculated based on the SPR of the two lowest SES groups combined, and the two highest SES groups combined, respectively. These SES groups were combined to provide a more stable estimation of the rate difference and the rate ratio between the lower and higher SES groups, respectively. [27] It complements the RII, as the latter takes into account all SES groups separately.

The relative index inequality (RII) was used to estimate the magnitude of inequalities in healthcare utilisation in a more comprehensive way. The RII is a regression-based index that assesses the

For peer review only - http://bmjopen.bmj.com/site/about/guidelines.xhtml

probability of healthcare use in relationship to the relative hierarchical position of every individual within the socioeconomic hierarchy. A higher RII indicates a stronger association between this hierarchical position and healthcare utilisation and implies a greater difference in utilisation between higher SES groups compared to lower SES groups. More specifically, RII=1 indicates equality, RII<1 indicates higher utilisation among lower SES, and RII>1 indicates higher utilisation among higher SES. Details on how RII calculated can be found elsewhere.[28] RII has property to estimate the magnitude of inequalities in one single measure that uses information from all socioeconomic groups individually and allows comparison between different socioeconomic and outcome indicator. RII is commonly used in epidemiological research and relatively has relatively a straightforward interpretation for readers who have no economics background compared to other common inequality measurements such as concentration index. The regression model was adjusted for age, sex and healthcare need, by controlling for SAH in the final model.

To correct for attrition and oversampling, the study sample was weighted with individual weights provided by the IFLS5. We used IBM SPPS Statistics 24 as statistical package to analyse the data.

Patient and public involvement

No patients were involved in this study; members of the public were not directly involved in this study.

RESULTS

The study sample included slightly more female respondents (51.6%) than males (Table 1). Almost two-thirds of the respondents were aged 15-45 years. Males had a generally higher level of education as compared to females. Primary care was the most frequently used type of healthcare, with 14.6% of the respondents reporting that they utilised primary care at least once in the previous four weeks. The highest utilisation of preventive care was for blood pressure measurement, with 80.5% of the respondents reporting that their blood pressure was measured during the previous 12 months.

For peer review only - http://bmjopen.bmj.com/site/about/guidelines.xhtml

The prevalence rates of primary care use were about similar across all educational levels (Table 2, see also online supplementary file Figure 1). Outpatient secondary care utilisation was more frequent among people with a higher educational level compared to people with a lower educational level. For overall inpatient care utilisation, the prevalence rates gradually increased with increasing educational level. A linear association was found between healthcare utilisation and income quintiles for all types of healthcare. This association was particularly strong for utilisation of outpatient secondary care and inpatient care.

Table 3 (see also online supplementary file Figures 2 and 3) quantifies the magnitude of educational and income inequalities in the utilisation of healthcare. Our findings from simple inequality measurement (rate ratio and rate difference) showed similarities with the findings from sophisticated inequality measurement (RII). No educational inequalities were found in primary care utilisation in the crude analysis, but positive educational inequalities (i.e. higher education associated with higher use rates) emerged after adjusting for SAH (RII 1.13, 95% CI 1.01-1.26). We consistently found positive educational inequalities in all types and levels of health care use after adjusting for SAH. The largest educational inequality was found in outpatient secondary care utilisation (RII 10.35, 95% CI 8.11-13.22).

Positive income inequalities (i.e. higher income associated with higher use rates) were found in all types and levels of healthcare use, especially after adjustment for SAH. Similar to educational inequalities, the largest income inequality was found in outpatient secondary care utilisation (RII 7.43, 95% CI 5.88-9.39). Generally, larger inequalities were found in relationship to income as compared to educational level, except for utilisation of outpatient secondary care.

A consistent linear association was found between prevalence rate of preventive care utilisation and SES (Table 4, and online supplementary file Figure 1). The prevalence rate of blood pressure measurement increased incrementally by SES group for both educational level and income quintiles. The prevalence rate of cholesterol tests, blood glucose tests and ECG tests drastically increased from the

For peer review only - http://bmjopen.bmj.com/site/about/guidelines.xhtml

third highest SES groups to the highest SES groups, both for income and educational level. The differences were larger in relationship to educational level than to income.

Table 5 shows the estimates of the size socioeconomic inequalities in preventive care utilisation (see also online supplementary file Figures 2 and 3). Our analyses showed consistent findings between simple (rate difference and rate ratio) and sophisticated inequality estimations (RII). Exceptionally large positive educational inequalities were found in blood glucose tests (RII 30.31, 95% CI 26.13-35.15) and ECG tests (RII 30.90, 95% CI 24.97-38.23). For income inequalities, inequalities in preventive care utilisation were smaller compared to educational inequalities. ECG tests showed the largest income inequality (RII 12.96, 95% CI 10.68-15.73), and blood pressure measurements showed the smallest inequality (RII 3.40, 95% CI 3.04-3.79).

DISCUSSION

This study documented socioeconomic inequalities in healthcare utilisation among the adult population in Indonesia. These inequalities were particularly large for secondary and preventive care. Compared to educational inequalities, income-related inequalities were larger for primary care and inpatient care, but smaller for outpatient secondary and preventive care.

This study was based on a nationally representative survey with a high response rate (95.3%) and with measurements that matched established international standards.[29] A possible limitation of the study is the measurement of healthcare need, that was limited to SAH. Ideally, we would have used multiple measures of healthcare need, such as self-reported morbidities or health functioning. Although our dataset provided self-reported morbidities and data on health functioning, these are likely to be underestimated in the Indonesian population (particularly in lower SES groups)[30], and therefore invalid for healthcare need adjustments.

For peer review only - http://bmjopen.bmj.com/site/about/guidelines.xhtml

Because no registry-based data on inequalities in healthcare utilisation in Indonesia are available, we used self-reported use of healthcare. Such healthcare utilisation measures may be subject to recall bias. However, the problem of recall bias might be limited, as the prevalence values of outpatient and inpatient care utilisation from the IFLS5 are close to the national average in Indonesia as reported by the Ministry of Health and data from the National Economic Survey.[31 32]

Previous studies in Indonesia mostly focus on specific healthcare services such as maternal and child-related healthcare. Our findings show that the direction and magnitude of inequalities in healthcare use among individuals aged 15 years or older bear resemblance to the large socioeconomic inequalities in maternal healthcare and child healthcare.[18 22] Similar to the recent study on wealth-related inequality in healthcare utilisation in Indonesia, we found smaller inequalities in the utilisation of primary care, especially outpatient care, and larger inequalities in secondary care.[23] Our results are also consistent with studies performed in other LMICs showing relatively small inequalities in PC utilisation, and larger inequalities in secondary care.[7-10]

The small socioeconomic inequalities in primary care utilisation are probably related to the relatively high supply and geographical distribution of primary care providers in Indonesia. Of all registered physicians in Indonesia, 78.4% are general practitioners that mostly practice as public or private providers. In total, there are 9745 public primary care centres providing services for the national population with subsidy by local governments.[33 13] Moreover, according to recent studies, access to primary care was increased by a government-financed NHI program that aimed to reduce financial barriers of the poor population to healthcare.[32 34 23] In the NHI program, primary care acted as gatekeeper which required all the beneficiaries regardless of their socioeconomic background (poor people or government employee) to use primary care as an entry point to access the healthcare service.[35] For people without insurance coverage, primary care is relatively affordable and can be accessed at low cost, even in private practices.[13] This likely explained the smaller income and

For peer review only - http://bmjopen.bmj.com/site/about/guidelines.xhtml

educational-related inequalities in the primary care utilisation compared to the inequalities in secondary and inpatient care utilisation.

In contrast to primary care, the use of secondary care facilities in Indonesia showed considerable inequalities by both educational level and income. For example, individuals with the highest income had seven times higher odds to use outpatient secondary care compared to those with the lowest income. It is likely that geographical barriers contribute to these inequalities. Because most secondary care facilities and specialists are located in urban areas, the poor need to pay high indirect costs (in terms of travel and opportunity) to access secondary care, even if their medical costs are covered by the NHI program.[23 32 34] Moreover, there is a limited supply of secondary care specialists; these specialists tend to work in private for-profit healthcare providers, which are not contracted by the NHI program. This is likely to result in low financial access for lower SES groups rather than higher SES groups, which may have supplementary private health insurance.[18 21]

We observed inequalities to be larger outpatient secondary care than for inpatient care. A possible explanation is that outpatient secondary care is much more affordable for higher income groups than for lower groups, as the former can pay the service by out-of pocket payment or private health insurance. Lower income groups generally can use outpatient secondary care only by using the government health insurance with its referral system. For inpatient care, however, utilisation costs are significant for higher income groups as well as lower income groups, and usually only affordable via government health insurance and accessible through a referral system.[21]

Inefficient referral procedures could also have contributed to larger inequalities in secondary care utilisation compared to primary care particularly for educational-related inequalities. Even when low-educated people are entitled to access secondary healthcare, they may lack the knowledge required to obtain a referral, due to the complexity of the administrative procedures in the referral system.[34] Inequalities in secondary care may also be influenced by differences between educational groups in the

For peer review only - http://bmjopen.bmj.com/site/about/guidelines.xhtml

preferences and resources that influence the way people utilise healthcare.[36] An Indonesian study showed that patients with higher educational level, regardless of their income level, were more likely to judge the quality of primary care to be low and to ask for a referral to secondary care. This tendency was not observed among people with high income, but relatively low education.[37 38] Education-related preferences might explain why educational inequalities in outpatient secondary care were larger compared to income-related inequalities.

We observed exceptionally large socioeconomic inequalities in preventive care, particularly by education. For example, individuals who had the highest educational level had 30 times higher odds to have a blood glucose test in the previous 12 months compared to those who had the lowest educational level. The individual's level of health literacy may play a major role in their use of preventive care.[39] Those with a relatively low level of health literacy may experience cognitive barriers to make decisions regarding diagnostic tests and treatments that they may need, irrespective of financial, geographic or administrative barriers.[40 41] This also likely explains relatively smaller educational-related inequalities in blood pressure measurement compared to other types of preventive care because blood pressure disorder such as high blood pressure is relatively known by common people regardless their educational background compared to other types of preventive care

The exceptionally large inequalities in preventive care utilisation may reflect the low priority given to preventive care in Indonesia's health policy which to date has strongly focused on curative care. This resulted in low health expenditures on preventive care[42], and the absence of a nationwide preventive program for the NCDs. As a result, the utilisation of preventive care is relying more on personal resources or potentially motivated or initiated by physicians who have more attention to preventive care.[43 44]

For peer review only - http://bmjopen.bmj.com/site/about/guidelines.xhtml

CONCLUSIONS

The findings underline the need to develop comprehensive efforts to tackle significant socioeconomic inequalities in healthcare utilisation in Indonesia. Potential areas of priority include removing financial and geographical barriers by providing the NHI program with universal health coverage, improving the supply and distribution of secondary care services, simplifying the referral system procedure, and developing a nationwide preventive care program. Improving the quality of primary care by providing better infrastructure and developing the competence of health personnel may have large impact on population health considering the (equality in) accessibility of primary care, and could potentially reduce the burden of secondary care. Monitoring healthcare (in)equality will be essential to evaluate the impact of these policies. Further research is needed to assess inequalities in healthcare among specific patient groups, and to evaluate the contribution of patient preferences and resources, and to examine the role of geographical factors and healthcare organisation and infrastructure. Such in-depth analyses could provide a better understanding of socioeconomic inequalities in healthcare utilisation in Indonesia and guide the development of strategies to address those inequalities.

Acknowledgement The authors thank the RAND Corporation for providing the dataset of the Indonesian Family Life Survey (IFLS) for this study.

Author Contributions JM conceived the paper. JM and AEK developed the analysis strategy. JM conducted the data analysis. JM, DSK, AEK collectively interpreted the findings. JM prepared the initial draft of the manuscript. JM, DSK, AEK equally contributed to the revision of the manuscript. All authors have read and approved the final manuscript.

Funding This study is funded by the Indonesian Endowment Fund for Education (LPDP), Ministry of Finance, Republic of Indonesia, grant number 20160322045795. The funding source has no role in study design, data collection, data analysis and interpretation, manuscript writing, and decision to submit or publish.

Competing Interest None declared

Patient consent Not required

Ethics approval This study is a secondary analysis using the Indonesian Family Life Survey (IFLS) dataset. The IFLS was approved by the Institutional Review Board (IRB) of the Rand Corporation (USA) and the

For peer review only - http://bmjopen.bmj.com/site/about/guidelines.xhtml

For peer review only

Survey Meter (Indonesia). The data set is publicly available and no personal information can be identified. This study is categorised as being exempt from human research according to the National Institute of Health (NIH).

Provenance and peer review Not commissioned; externally peer reviewed.

Data sharing statement This study used the Indonesian Family Life Survey (IFLS) dataset provided by RAND Corp. The IFLS dataset (including the supporting documents such as survey protocol and questionnaire) is freely accessible at https://www.rand.org/labor/FLS/IFLS.html.

Open Access This is an Open Access article distributed in accordance with the Creative Commons Attribution Non Commercial (CC BY-NC 4.0) license, which permits others to distribute, remix, adapt, build upon this work non-commercially, and license their derivative works on different terms, provided the original work is properly cited and the use is non-commercial. See: http://creativecommons.org/licenses/by-nc/4.0/

REFERENCES

1. Oliver A, Mossialos E. Equity of access to health care: outlining the foundations for action. Journal of epidemiology and community health 2004;**58**(8):655-58.
2. Culyer AJ, Wagstaff A. Equity and equality in health and health care. Journal of health economics 1993;**12**(4):431-57.
3. Goddard M, Smith P. Equity of access to health care services: theory and evidence from the UK. Soc Sci Med 2001;**53**.
4. van Doorslaer E, Masseria C, Koolman X. Inequalities in access to medical care by income in developed countries. Cmaj 2006;**174**.
5. Walters S, Suhrcke M. Socioeconomic inequalities in health and health care access in central and eastern Europe and the CIS: a review of the recent literature. WHO European Office for Investment for Health and Development, 2005.
6. Balabanova D, McKee M, Pomerleau J. Health service utilization in the former soviet union: evidence from eight countries. Health Serv Res 2004;**39**.
7. Makinen M, Waters H, Rauch M, et al. Inequalities in health care use and expenditures: empirical data from eight developing countries and countries in transition. Bulletin of the World Health Organization 2000;**78**:55-65.
8. Zhang X, Wu Q, Shao Y, et al. Socioeconomic Inequities in Health Care Utilization in China. Asia Pacific Journal of Public Health 2015;**27**(4):429-38.
9. Brinda EM, Attermann J, Gerdtham UG, et al. Socio-economic inequalities in health and health service use among older adults in India: results from the WHO Study on Global AGEing and adult health survey. Public Health 2016;**141**:32-41.
10. Yiengprugsawan V, Carmichael G, Lim LY, et al. Explanation of inequality in utilization of ambulatory care before and after universal health insurance in Thailand. Health policy and planning 2011;**26**(2):105-14.
11. O'Donnell O. Access to health care in developing countries: breaking down demand side barriers. Cadernos de saude publica 2007;**23**(12):2820-34.
12. Peters DH, Garg A, Bloom G, et al. Poverty and access to health care in developing countries. Annals of the New York Academy of Sciences 2008;**1136**:161-71.

For peer review only - http://bmjopen.bmj.com/site/about/guidelines.xhtml

13. Mahendradhata Y, Trisnantoro L, Listyadewi S, et al. The Republic of Indonesia Health System Review. In: Hort K, Patcharanarumol W, eds. Health Systems in Transition. India: World Health Organization, Regional Office for South-East Asia, 2017.
14. Mboi N. Indonesia: On the Way to Universal Health Care. Health Systems & Reform 2015;**1**(2):91-97.
15. Pisani E, Olivier Kok M, Nugroho K. Indonesia's road to universal health coverage: a political journey. Health policy and planning 2017;**32**(2):267-76.
16. Agustina R, Dartanto T, Sitompul R, et al. Universal health coverage in Indonesia: concept, progress, and challenges. The Lancet 2019;**393**(10166):75-102.
17. Widjaja FF. Universal health coverage in Indonesia – the forgotten prevention. 2014 2014;**23**(3):-118 %J Medical Journal of Indonesia.
18. Barber SL, Gertler PJ, Harimurti P. Differences In Access To High-Quality Outpatient Care In Indonesia. Health Affairs 2007;**26**(3):w352-w66.
19. Maharani DA. Inequity in dental care utilization in the Indonesian population with a self-assessed need for dental treatment. The Tohoku journal of experimental medicine 2009;**218**(3):229-39.
20. Maharani DA, Rahardjo A. Is the utilisation of dental care based on need or socioeconomic status? A study of dental care in Indonesia from 1999 to 2009. International dental journal 2012;**62**(2):90-4.
21. Utomo B, Sucahya PK, Utami FR. Priorities and realities: addressing the rich-poor gaps in health status and service access in Indonesia. International Journal for Equity in Health 2011;**10**(1):47.
22. Hosseinpoor AR, Bergen N, Floranita R, et al. State of health inequality: Indonesia. Geneva: World Health Organization, 2017.
23. Johar M, Soewondo P, Pujisubekti R, et al. Inequality in access to health care, health insurance and the role of supply factors. Social Science & Medicine 2018;**213**:134-45.
24. Strauss J, Witoelar F, Sikoki B. The Fifth Wave of the Indonesia Family Life Survey: Overview and Field Report: RAND Labor and Population, 2016.
25. O'Donnel O, Doorslaer Ev, Wagstaff A, et al. *Analyzing Health Equity Using Household Survey Data: A Guide to Techniques and Their Implementation*. Washington: The World Bank Institute, 2008.
26. Riley LM, Cowan M. Noncommunicable Diseases Country Profile 2014. Geneva: World Health Organization, 2014.
27. Hosseinpoor AR, Bergen N. Handbook on Health Inequality Monitoring with a special focus in low-middle income countries Geneva: Worrld Health Organization, 2013.
28. Mackenbach JP, Kunst AE. Measuring the magnitude of socio-economic inequalities in health: an overview of available measures illustrated with two examples from Europe. Soc Sci Med 1997;**44**(6):757-71.
29. Galobardes B, Lynch J, Smith GD. Measuring socioeconomic position in health research. British Medical Bulletin 2007;**81-82**(1):21-37.
30. Vellakkal S, Millett C, Basu S, et al. Are estimates of socioeconomic inequalities in chronic disease artefactually narrowed by self-reported measures of prevalence in low-income and middle-income countries? Findings from the WHO-SAGE survey. Journal of epidemiology and community health 2014:jech-2014-204621.
31. *Riset Kesehatan Dasar 2013*. Jakarta: Badan Penelitian dan Pengembangan Kementerian Kesehatan Republik Indonesia, 2013.
32. Sparrow R, Suryahadi A, Widyanti W. Social health insurance for the poor: Targeting and impact of Indonesia's Askeskin programme. Social Science & Medicine 2013;**96**:264-71.
33. Profil Kesehatan Indonesia 2015. Jakarta: Kementerian Kesehatan Republik Indonesia, 2016.
34. Harimurti P, Pambudi E, Pigazzini A, et al. The Nuts & Bolts of Jamkesmas, Indonesia's Government-financed Health Coverage Program for the Poor and Near-Poor. UNICO Studies Series. Washington DC: The World Bank, 2013.

For peer review only - http://bmjopen.bmj.com/site/about/guidelines.xhtml

35. Hidayat B, Thabrany H, Dong H, et al. The effects of mandatory health insurance on equity in access to outpatient care in Indonesia. Health Policy and Planning 2004;**19**(5):322-35.
36. Levesque J-F, Harris MF, Russell G. Patient-centred access to health care: conceptualising access at the interface of health systems and populations. International Journal for Equity in Health 2013;**12**:18-18.
37. Istiono W, Claramita M, Ekawati FM, et al. Physician's self-perceived abilities at primary care settings in Indonesia. Journal of Family Medicine and Primary Care 2015;**4**(4):551-58.
38. Sari MI, Prabandari YS, Claramita M. Physicians' professionalism at primary care facilities from patients' perspective: The importance of doctors' communication skills. Journal of Family Medicine and Primary Care 2016;**5**(1):56-60.
39. Gottfredson LS. Intelligence: is it the epidemiologists' elusive" fundamental cause" of social class inequalities in health? Journal of personality and social psychology 2004;**86**(1):174.
40. Chen JZ, Hsu HC, Tung HJ, et al. Effects of health literacy to self-efficacy and preventive care utilization among older adults. Geriatrics & gerontology international 2013;**13**(1):70-6.
41. Huang C, Liu CJ, Pan XF, et al. Correlates of unequal access to preventive care in China: a multilevel analysis of national data from the 2011 China Health and Nutrition Survey. BMC Health Serv Res 2016;**16**:177.
42. Widjaja FF. Universal health coverage in Indonesia–the forgotten prevention. Med J Indones 2014;**23**(3).
43. Rodin D, Stirbu I, Ekholm O, et al. Educational inequalities in blood pressure and cholesterol screening in nine European countries. J Epidemiol Community Health 2012;**66**(11):1050-5.
44. Palència L, Espelt A, Rodríguez-Sanz M, et al. Socio-economic inequalities in breast and cervical cancer screening practices in Europe: influence of the type of screening program. International Journal of Epidemiology 2010;**39**(3):757-65.

For peer review only

For peer review only - http://bmjopen.bmj.com/site/about/guidelines.xhtml

Table 1. Basic characteristics of the study population.

Variables	Total n	Total %	Male n	Male %	Female n	Female %
**Gender**						
Male	20374	48.4	-	-	-	-
Female	21709	51.6	-	-	-	-
**Age group (in years)**						
15-30	12471	29.6	6436	31.6	6035	27.8
31-45	14049	33.4	6545	32.1	7503	34.6
46-60	10280	24.4	4973	24.4	5306	24.4
>60	5283	12.6	2419	11.9	2864	13.2
**Education level**						
Pre-primary	9868	23.4	3977	19.5	5891	27.1
Primary	9993	23.7	4855	23.8	5138	23.7
Lower secondary	8082	19.2	4041	19.8	4041	18.6
Upper secondary	10731	25.2	5894	28.9	4838	22.3
Tertiary	3409	8.1	1607	7.9	1802	8.3
**Income[a]**						
1st quintile (230-1300)	8417	20.0	4050	19.9	4367	20.1
2nd quintile (1300-1830)	8418	20.0	3997	19.6	4421	20.4
3rd quintile (1830-2520)	8415	20.0	4056	19.9	4359	20.1
4th quintile (2520-3830)	8417	20.0	4159	20.4	4258	19.6
5th quintile (3830-55400)	8416	20.0	4111	20.2	4305	19.8
**Self-assessed health**						
Very healthy	8137	19.3	4362	21.4	3775	17.4
Somewhat healthy	24757	58.8	12179	59.8	12578	57.9
Somewhat unhealthy	8447	20.1	3513	17.2	4934	22.7
Very unhealthy	742	1.8	320	1.6	422	1.9
**Outpatient care utilisation**						
Primary care	6155	14.6	2006	9.8	4149	19.1
Secondary care	1022	2.4	427	2.1	595	2.7
Total	6864	16.3	2323	11.4	4541	20.9
**Inpatient care utilisation**						
Overall	1937	4.6	591	2.9	1346	6.2
**Preventive care utilisation (age ≥ 31 years)**						
Blood pressure screening	21663	80.5	9254	74.3	12409	85.4
Cholesterol screening	4678	17.4	1951	15.7	2727	18.9
Blood glucose screening	4142	15.4	1855	14.9	2287	15.8
Electrocardiography test	1723	6.4	876	7.0	847	5.9

[a]Income in thousands Indonesian Rupiah (IDR)

For peer review only

For peer review only - http://bmjopen.bmj.com/site/about/guidelines.xhtml

Table 2. Standardised prevalence rate (SPR) of healthcare utilisation by socioeconomic status.

	Outpatient care (SPR, 95% CI)†			Inpatient care (SPR, 95% CI)
	Primary	Secondary	Total	Overall
**Education**				
Pre-primary	14.47(13.78-15.18)	1.31(1.12-1.53)	15.22(14.52-15.95)	3.07(2.77-3.40)
Primary	14.93(14.19-15.70)	2.03(1.76-2.32)	16.07(15.30-16.87)	4.60(4.18-5.04)
Lower secondary	15.00(14.12-15.91)	1.88(1.57-2.22)	16.38(15.47-17.33)	4.31(3.86-4.81)
Upper secondary	14.38(13.64-15.16)	3.39(3.03-3.79)	16.99(16.18-17.84)	6.12(5.63-6.64)
Tertiary	14.37(13.12-15.71)	6.26(5.44-7.17)	18.97(17.53-20.50)	6.93(6.04-7.91)
**Income**				
1st quintile	11.71(11.02-12.42)	1.32(1.09-1.57)	12.54(11.83-13.29)	2.99(2.65-3.35)
2nd quintile	14.11(13.34-14.92)	1.44(1.20-1.71)	14.73(13.94-15.55)	3.76(3.37-4.18)
3rd quintile	15.36(14.54-16.22)	2.00(1.71-2.33)	16.75(15.89-17.64)	4.24(3.81-4.70)
4th quintile	15.73(14.88-16.62)	2.91(2.55-3.31)	18.57(17.64-19.53)	5.13(4.64-5.65)
5th quintile	16.13(15.24-17.06)	4.97(4.48-5.50)	19.83(18.74-20.85)	7.56(6.94-8.21)

†Prevalence rate per 100 persons, age and sex standardised to the total population.

Table 3. Socioeconomic inequalities in the utilisation of various types and levels of healthcare.

	Type of care	Level of care	SPR (95% CI)†		Rate difference	Rate ratio	RII (95% CI), adjusted for age, sex	RII (95% CI), adjusted for age, sex, SAH
			Two lowest groups	Two highest groups				
Education	Outpatient	Primary	14.68(14.18-15.20)	14.38(13.74-15.05)	-0.30	0.98	0.99 (0.98-1.01)	1.13 (1.01-1.26)
		Secondary	1.64(1.47-1.82)	2.88 (2.59-3.19)	1.24	1.76	7.89 (6.33-9.85)	10.35 (8.11-13.22)
		Total	15.62(15.09-16.15)	17.51(16.80-18.25)	1.89	1.12	1.35 (1.24-1.46)	1.59 (1.44-1.77)
	Inpatient	Overall	3.74(3.48-4.01)	6.31(5.88-6.76)	2.57	1.69	2.38 (1.97-2.76)	2.78 (2.32-3.32)
Income	Outpatient	Primary	12.88(12.35-13.42)	16.25(15.62-16.89)	3.37	1.26	1.50 (1.39-1.62)	1.68 (1.52-1.85)
		Secondary	1.36(1.20-1.54)	4.69(4.35-5.04)	3.33	3.45	6.61 (5.29-8.25)	7.43 (5.88-9.39)
		Total	13.61(13.08-14.16)	19.18(18.50-19.88)	5.57	1.41	1.80 (1.67-1.94)	2.15 (1.96-2.36)
	Inpatient	Overall	3.36(3.10-3.63)	6.30(5.91-6.71)	2.94	1.88	2.94 (2.52-3.43)	3.11 (2.63-3.66)

†Prevalence rate per 100 persons, age and sex standardised to the total population.

For peer review only - http://bmjopen.bmj.com/site/about/guidelines.xhtml

Table 4. Standardised prevalence rate (SPR) of preventive care utilisation by socioeconomic status.

	Preventive care activity (SPR, 95% CI)†			
	Blood pressure	Cholesterol	Blood glucose	ECG
**Education**				
Pre-primary	72.40(70.54-74.30)	8.85(8.24-9.49)	6.85(6.32-7.41)	2.51(2.19-2.86)
Primary	79.54(77.47-81.65)	10.78(10.03-11.58)	10.59(9.84-11.39)	4.00(3.54-4.50)
Lower secondary	82.72(79.88-85.63)	18.03(16.67-19.48)	15.44(14.17-16.79)	4.95(4.25-5.73)
Upper secondary	86.66(84.27-89.10)	26.83(25.44-28.27)	24.72(23.39-26.10)	10.80(9.94-11.71)
Tertiary	92.33(88.45-96.35)	44.72(41.97-47.60)	43.38(40.66-46.23)	20.99(19.13-22.99)
**Income**				
1st quintile	73.55(71.27-75.88)	7.83(7.11-8.60)	6.12(5.49-6.81)	2.71(2.29-3.18)
2nd quintile	76.77(74.45-79.15)	12.12(11.21-13.08)	10.14(9.30-11.02)	3.37(2.90-3.90)
3rd quintile	80.60(78.22-83.03)	14.37(13.37-15.42)	12.63(11.70-13.63)	4.64(4.08-5.26)
4th quintile	83.68(81.25-86.16)	20.34(19.14-21.59)	18.41(17.27-19.60)	7.13(6.43-7.89)
5th quintile	87.87(85.38-90.41)	32.69(31.17-34.26)	30.09(28.64-31.60)	14.43(13.43-15.49)

†Prevalence rate per 100 persons, age and sex standardised to the total population

Table 5. Socioeconomic inequalities in the use of various preventive care activities.

	Activity	SPR (95% CI)†		Rate difference	Rate ratio	RII (95% CI) adjusted for age, sex	RII (95% CI) adjusted for age, sex, SAH
		Two lowest groups	Two highest groups				
**Education**	Blood pressure	75.77(74.38-77.17)	88.28(86.24-90.36)	12.51	1.17	6.37 (5.61-7.24)	6.67(5.87-7.59)
	Cholesterol	9.69(9.22-10.19)	32.12(30.85-33.44)	22.43	3.31	18.17(15.91-20.74)	21.27(18.53-24.41)
	Blood glucose	8.47(8.03-8.94)	30.21(28.98-31.49)	21.74	3.57	24.61(21.36-28.36)	30.31(26.13-35.15)
	ECG	3.17(2.90-3.46)	13.74(12.92-14.60)	10.57	4.33	25.45(20.72-31.20)	30.90(24.97-38.23)
**Income**	Blood pressure	75.17(73.54-76.83)	86.28(84.54-88.05)	11.11	1.15	3.34(2.99-3.72)	3.40(3.04-3.79)
	Cholesterol	8.61(8.06-9.17)	26.54(25.57-25.53)	17.93	3.08	9.20(8.15-10.40)	9.76 (8.63-11.02)
	Blood glucose	8.11(7.59-8.66)	24.29(23.36-35.54)	16.18	3.00	10.81(9.49-12.30)	11.59(10.18-13.20)
	ECG	3.03(2.71-3.37)	10.78(10.14-11.40)	7.73	3.55	12.42(10.23-15.07)	12.96(10.68-15.73)

†Prevalence rate per 100 persons, age and sex standardised to the total population

For peer review only - http://bmjopen.bmj.com/site/about/guidelines.xhtml

Table 1. Distribution of self-assessed health status (SAH) among different socioeconomic status (SES)

	Self-assessed health (SAH)			
	Very healthy n (%)	Somewhat healthy n (%)	Somewhat unhealthy n (%)	Very unhealthy n (%)
**Educational level**				
Pre-primary	1900 (19.3)	4922 (49.9)	2693 (27.3)	353 (3.6)
Primary	1980 (19.8)	5652 (56.6)	2178 (21.8)	182 (1.8)
Lower secondary	1538 (19.0)	4956 (61.3)	1499 (18.5)	89 (1.1)
Upper secondary	2044 (19.0)	6957 (64.8)	1641 (15.3)	90 (0.8)
Tertiary	675 (19.8)	2270 (66.6)	436 (12.8)	27 (0.8)
**Income**				
1st quintile (poorest)	1683 (18.8)	5031 (56.3)	2036 (22.8)	194 (2.2)
2nd quintile	1637 (18.9)	5077 (58.5)	1778 (20.5)	188 (2.2)
3rd quintile	1649 (19.4)	5081 (59.8)	1619 (19.1)	143(1.7)
4th quintile	1622 (19.8)	4894 (59.7)	1570 (19.2)	112 (1.4)
5th quintile (richest)	1544 (19.9)	4674 (60.2)	1444 (18.6)	105 (1.4)

For peer review only

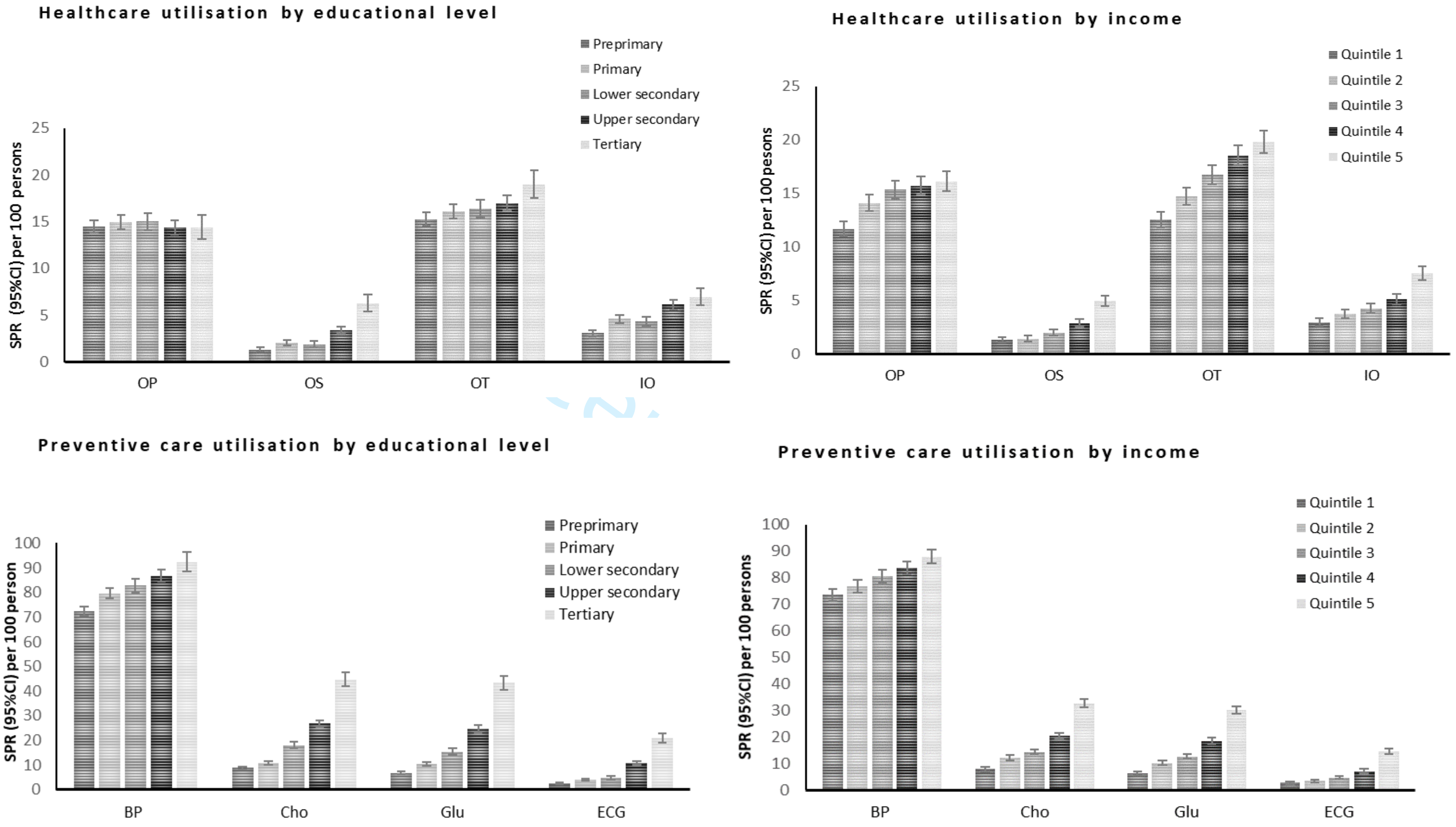

Figure 1. Standardised prevalence rate (95%CI) for healthcare and preventive care utilisation. Prevalence rate is per 100 persons, standardised by age and sex to total population. OP: Outpatient primary care; OS: Outpatient secondary care; OT: Outpatient total; IO: Inpatient overall; BP: Blood pressure; Cho: Cholesterol; Glu: Blood glucose; ECG: Electrocardiograph.

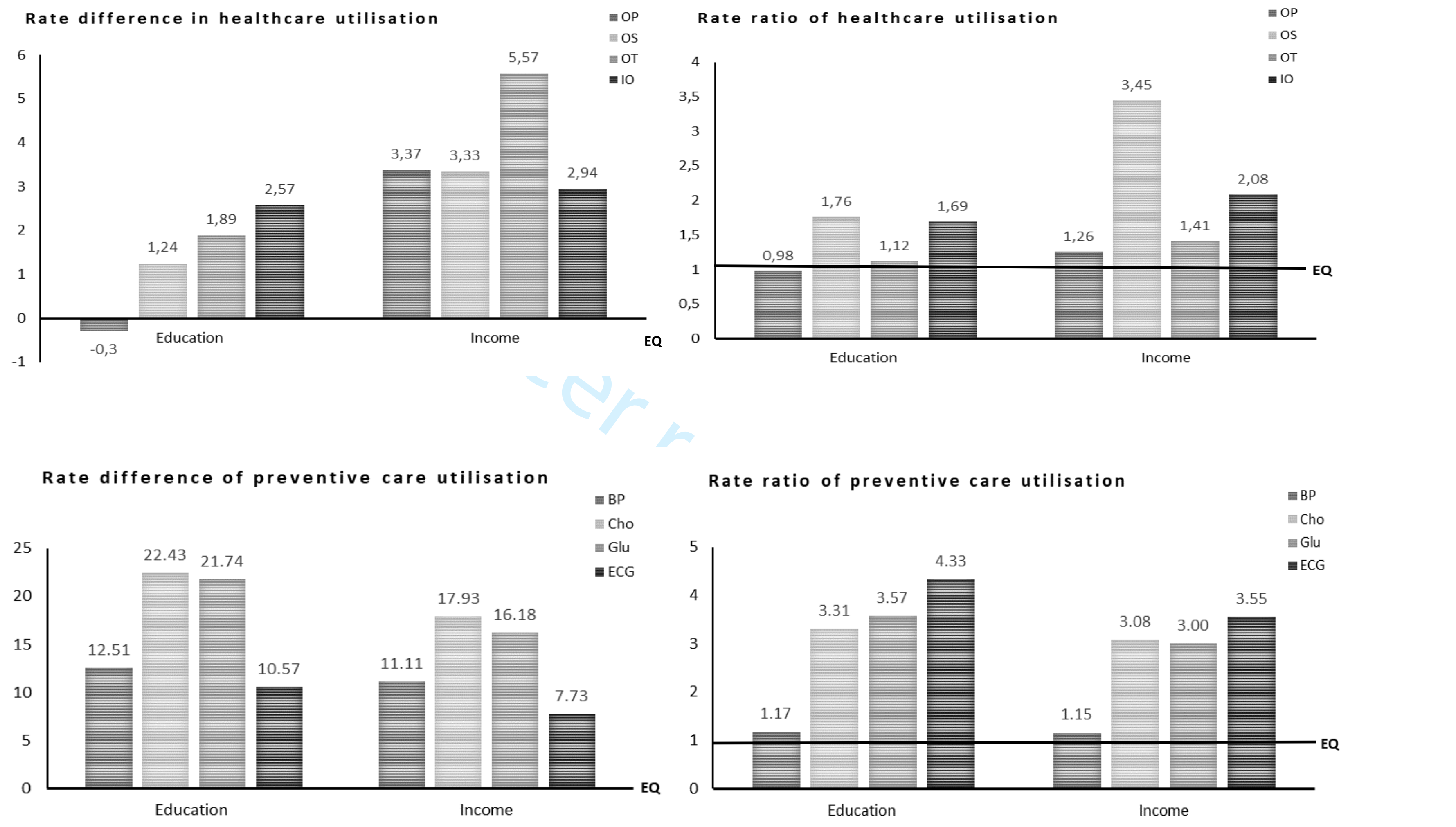

Figure 2. Simple measurement of absolute (rate difference) and relative (rate ratio) inequalities in healthcare and preventive care utilisation between two highest and two lowest groups of SES. Rate difference is per 100 persons. OP: Outpatient primary care; OS: Outpatient secondary care; OT: Outpatient total; IO: Inpatient overall; BP: Blood pressure; Cho: Cholesterol; Glu: Blood glucose; ECG: Electrocardiograph; EQ: Equality line.

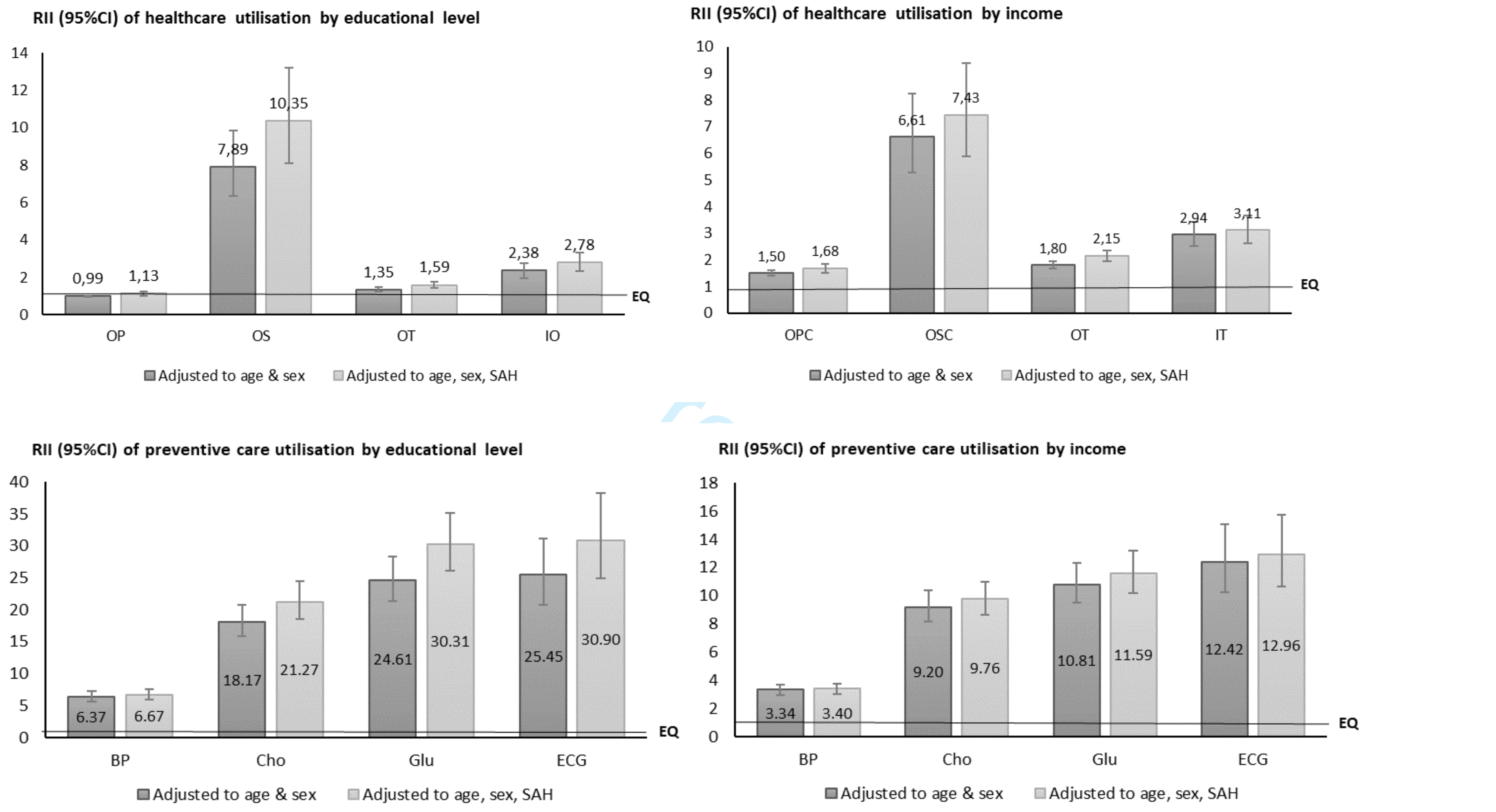

Figure 3. Relative index inequality (95%CI) of healthcare and preventive care utilisation by educational level and income. OP: Outpatient primary care; OS: Outpatient secondary care; OT: Outpatient total; IO: Inpatient overall; BP: Blood pressure; Cho: Cholesterol; Glu: Blood glucose; ECG: Electrocardiograph; EQ: Equality line; SAH: Self-assessed health

STROBE Statement—Checklist of items that should be included in reports of ***cross-sectional studies***

	Item No	Recommendation
**Title and abstract**	1	(*a*) Indicate the study's design with a commonly used term in the title or the abstract **[see methods section of abstract page 2]**
		(*b*) Provide in the abstract an informative and balanced summary of what was done and what was found **[see methods and results section of abstract page 2]**
**Introduction**		
Background/rationale	2	Explain the scientific background and rationale for the investigation being reported **[page 4-5]**
Objectives	3	State specific objectives, including any pre-specified hypotheses [**page 6]**
**Methods**		
Study design	4	Present key elements of study design early in the paper **[page 6]**
Setting	5	Describe the setting, locations, and relevant dates, including periods of recruitment, exposure, follow-up, and data collection **[page 6]**
Participants	6	(*a*) Give the eligibility criteria, and the sources and methods of selection of participants **[page 6]**
Variables	7	Clearly define all outcomes, exposures, predictors, potential confounders, and effect modifiers. Give diagnostic criteria, if applicable **[page 7]**
Data sources/ measurement	8*	For each variable of interest, give sources of data and details of methods of assessment (measurement). Describe comparability of assessment methods if there is more than one group **[page 7]**
Bias	9	Describe any efforts to address potential sources of bias **[page 6]**
Study size	10	Explain how the study size was arrived at **[page 6]**
Quantitative variables	11	Explain how quantitative variables were handled in the analyses. If applicable, describe which groupings were chosen and why **[page 7]**
Statistical methods	12	(*a*) Describe all statistical methods, including those used to control for confounding **[page 8-9]**
		(*b*) Describe any methods used to examine subgroups and interactions **[N/A]**
		(*c*) Explain how missing data were addressed **[page 5]**
		(*d*) If applicable, describe analytical methods taking account of sampling strategy **[page 9]**
		(*e*) Describe any sensitivity analyses **[N/A]**
**Results**		
Participants	13*	(a) Report numbers of individuals at each stage of study—eg numbers potentially eligible, examined for eligibility, confirmed eligible, included in the study, completing follow-up, and analysed **[N/A]**
		(b) Give reasons for non-participation at each stage **[N/A]**
		(c) Consider use of a flow diagram **[N/A]**
Descriptive data	14*	(a) Give characteristics of study participants (eg demographic, clinical, social) and information on exposures and potential confounders [**page 9, table 1 (page 19]**
		(b) Indicate number of participants with missing data for each variable of interest **[N/A]**
Outcome data	15*	Report numbers of outcome events or summary measures **[page 9-11, table 2,4 (page 20,21]**

For peer review only

Main results	16	(*a*) Give unadjusted estimates and, if applicable, confounder-adjusted estimates and their precision (eg, 95% confidence interval). Make clear which confounders were adjusted for and why they were included **[page 9-11, table 3,5 (page 20,21)]**
		(*b*) Report category boundaries when continuous variables were categorized **[N/A]**
		(*c*) If relevant, consider translating estimates of relative risk into absolute risk for a meaningful time period **[N/A]**
Other analyses	17	Report other analyses done—eg analyses of subgroups and interactions, and sensitivity analyses **[N/A]**
**Discussion**		
Key results	18	Summarise key results with reference to study objectives **[page 11]**
Limitations	19	Discuss limitations of the study, taking into account sources of potential bias or imprecision. Discuss both direction and magnitude of any potential bias **[page 11-12]**
Interpretation	20	Give a cautious overall interpretation of results considering objectives, limitations, multiplicity of analyses, results from similar studies, and other relevant evidence **[page 12-14]**
Generalisability	21	Discuss the generalisability (external validity) of the study results **[page 15]**
**Other information**		
Funding	22	Give the source of funding and the role of the funders for the present study and, if applicable, for the original study on which the present article is based **[declaration section, page 15]**

*Give information separately for exposed and unexposed groups.

Note: An Explanation and Elaboration article discusses each checklist item and gives methodological background and published examples of transparent reporting. The STROBE checklist is best used in conjunction with this article (freely available on the Web sites of PLoS Medicine at http://www.plosmedicine.org/, Annals of Internal Medicine at http://www.annals.org/, and Epidemiology at http://www.epidem.com/). Information on the STROBE Initiative is available at www.strobe-statement.org.

For peer review only

For peer review only - http://bmjopen.bmj.com/site/about/guidelines.xhtml

BMJ Open

Socioeconomic inequalities in healthcare utilisation in Indonesia: a comprehensive survey-based overview

Journal:	*BMJ Open*
Manuscript ID	bmjopen-2018-026164.R2
Article Type:	Research
Date Submitted by the Author:	30-Apr-2019

Complete List of Authors:	Mulyanto, Joko; Department of Public Health, Amsterdam UMC, University of Amsterdam, Amsterdam Public Health research institute; Department of Public Health and Community Medicine, Faculty of Medicine, Universitas Jenderal Soedirman<br>Kringos, Dionne Sofia; Department of Public Health, Amsterdam UMC, University of Amsterdam, Amsterdam Public Health research institute<br>Kunst, Anton; Department of Public Health, Amsterdam UMC, University of Amsterdam, Amsterdam Public Health research institute

**Primary Subject Heading**:	Health services research
Secondary Subject Heading:	Health policy
Keywords:	Socioeconomic, Inequalities, Access, Healthcare, Indonesia

SCHOLARONE™ Manuscripts

Socioeconomic inequalities in healthcare utilisation in Indonesia: a comprehensive survey-based overview

Joko Mulyanto[1,2], Dionne S Kringos[1], Anton E Kunst[1]

[1]Department of Public Health, Amsterdam UMC, University of Amsterdam, Amsterdam Public Health research institute, Amsterdam, the Netherlands.
[2]Department of Public Health and Community Medicine, Faculty of Medicine, Universitas Jenderal Soedirman, Purwokerto, Indonesia

Corresponding author
Joko Mulyanto, Department of Public Health, Amsterdam UMC, University of Amsterdam, PO Box 22660 1100 DD, Amsterdam, the Netherlands; Telephone: +31-20-5664642; j.mulyanto@amc.uva.nl

For peer review only

ABSTRACT

Objective Monitoring inequality in healthcare utilisation is essential to reduce persistent inequalities in health in lower-middle income countries. This study aimed to assess socioeconomic inequalities in the utilisation of primary care, secondary care, and preventive care in Indonesia.

Methods A cross-sectional study was conducted using data from the 2014 Indonesia Family Life Survey with a total of 42,083 adult participants. Socioeconomic status (SES) was measured by educational level and income. Healthcare utilisation was measured in: i) primary care, ii) outpatient in secondary care, iii) inpatient care, and iii) cardiovascular-related preventive care. The magnitude of inequalities was measured using the relative index of inequality (RII).

Results Small educational inequalities were found for primary care utilisation (RII 1.13, 95% CI 1.01-1.26). Larger educational inequalities were found for outpatient secondary care (RII 10.35, 95% CI 8.11-13.22) and inpatient care (RII 2.78, 95% CI 2.32-3.32). The largest educational inequalities were found for preventive care, particularly regarding blood glucose tests (RII 30.31, 95% CI 26.13-35.15) and electrocardiography tests (RII 30.90, 95% CI 24.97-38.23). Compared to educational inequalities, income inequalities were larger for primary care (RII 1.68, 95% CI 1.52-1.85) and inpatient care (RII 3.11, 95% CI 2.63-3.66), but not for outpatient secondary care and preventive care.

Conclusions Socioeconomic inequalities in healthcare utilisation in Indonesia are particularly large in secondary and preventive care. Therefore, it is recommended to prioritise policies focused on improving timely, geographical and financial access to secondary and preventive care for lower SES groups.

Keywords socioeconomic: inequalities; access; healthcare: Indonesia

For peer review only - http://bmjopen.bmj.com/site/about/guidelines.xhtml

ARTICLE SUMMARY

Strengths and limitations of this study

- This study was based on a nationally representative survey with a high response rate and with measurements that matched established international standards.
- Few studies have investigated inequalities in healthcare utilisation in Indonesia
- The measurement of healthcare need was limited to self-assessed health.
- The measurement of healthcare utilisation was based on self-reported data which might be subject to recall bias.

For peer review only

INTRODUCTION

Equal use of healthcare for equal need is essential to improve population health and is, therefore, an objective for most healthcare systems. Monitoring inequality in healthcare utilisation is essential to assess the performance of a healthcare system, and ultimately, to reduce persistent inequalities in health.[1] To monitor inequality in use in these terms, healthcare utilisation should be adjusted for self-assessed health or morbidities, as determinants of healthcare need.[2 3]

There is evidence of inequalities in healthcare utilisation in developed countries, despite universal healthcare coverage.[4] For example, both in Western and Eastern Europe, inequalities in healthcare utilisation exist for certain types of healthcare. In Eastern Europe, the rapid transition of the healthcare system since the late 1990s after the fall of Communism may have been conducive to large inequalities.[5 6]

Lower-middle income countries (LMICs) also experience inequalities in healthcare utilisation especially in secondary care, as shown by an international comparative study in Asia, Africa and Latin America[7]; and studies in China and India.[8 9] Significant inequalities in healthcare utilisation are also found in Thailand, despite universal healthcare coverage since 2005.[10] For several reasons, sizeable inequalities in healthcare utilisation may also exist in other LMICs. Many of LMICs are struggling to provide universal healthcare coverage, resulting in persisting financial barriers to access healthcare. Furthermore, inadequate supply and unequal geographic distribution of healthcare facilities cause greater barriers to the use of these facilities by people living in remote places and with limited resources. Moreover, large inequalities in the quality of healthcare that is received may result from poor stewardship, low financial investments in the healthcare system, and suboptimal quality of a broad range of healthcare services.[11 12]

Indonesia is an LMIC with a population of 262 million people that are distributed across ≥17,000 islands, and with diverse ethnic and religious backgrounds. Indonesia's healthcare system is a mixture of public and private healthcare delivery systems. The size and role of the private-commercial healthcare

For peer review only - http://bmjopen.bmj.com/site/about/guidelines.xhtml

market have increased during the last decade. Total health expenditure in 2014 was 2.8% of the gross domestic product, of which 47% originated from out-of-pocket payments. Since 1999, the government has provided health insurance for the poor, and in 2014 introduced the National Health Insurance (NHI) program to remove financial barriers to access basic healthcare services for the entire population by 2019.[13]

Current policy to achieve equal access in healthcare in Indonesia is focusing on the expansion of the NHI program.[14] However, over the years, progress towards universal health coverage has been uneven and iterative and consistently driven by domestic political interests as opposed to technical considerations.[15] The dominance of political interest is also reflected in the government evaluation of the NHI program which emphasised the overall coverage (NHI membership) of the population and paid less attention to the issue of the actual access distribution such as inequality among various population groups.[16]

In terms of preventive care, communicable diseases are still the government's priority with the improvement of universal child immunisation as the main focus.[16] Until recently, Indonesia did not implement a systematic policy or programs for the prevention of cardiovascular diseases or other main non-communicable diseases (NCDs). [13] Furthermore, the NHI program put much emphasis on curative care, which makes the utilisation of preventive care likely depend more on personal resources than on collective efforts.[17]

Lack of information which comprehensively assess the current situation of inequalities in healthcare utilisation in Indonesia may contribute to the low attention of the government in this issue. During the last decade, only a few studies have investigated inequalities in healthcare utilisation in Indonesia. Previous studies focused on the inequalities in maternal and child-related healthcare and dental care.[18-21] A recent report from the WHO stated that large inequalities in maternal and child healthcare persist in Indonesia, in addition to geographic inequalities in the healthcare infrastructure,

For peer review only

For peer review only - http://bmjopen.bmj.com/site/about/guidelines.xhtml

particularly between the different provinces.[22] A recent study showed wealth-related inequalities in Indonesia in the use of healthcare, particularly in secondary care. However, this study did not assess inequalities in relation to other SES indicators such as educational level, nor did it consider inequalities in preventive care utilisation.[23]

No studies have empirically assessed socioeconomic inequalities (in terms of both educational level and income) in general healthcare utilisation in Indonesia particularly for preventive care utilisation. The present study aimed to fill in this gap of evidence. Using a large-scale national interview survey, we aimed to provide a comprehensive overview of socioeconomic inequalities in the utilisation of primary care, secondary care and preventive care in Indonesia. Findings from this study would be particularly beneficial for policymakers to assess the progress of the current efforts to reduce inequalities and also for policy development to further address inequalities in healthcare utilisation in Indonesia.

METHODS

Study design and data sources

We conducted a cross-sectional study using data from the fifth wave of the Indonesia Family Life Survey (IFLS5) which was conducted in 2014 by the RAND Corporation (USA). The IFLS5 is a longitudinal survey which has been conducted since 1993 (IFLS1) and collected data from 13 selected Indonesian provinces to maximally capture the diversity in the socioeconomic and cultural background of the Indonesian population. These 13 provinces represented 83% of the Indonesian population. The IFLS used stratified random sampling based on province and rural/urban location. The sampling frame was randomly chosen from the list enumeration area (EA) of the National Socioeconomic Survey which was conducted by the National Bureau of Statistics in more than 60,000 households. Within each urban EA, 20 households were randomly selected while 30 households were selected from each rural EA. In total, 7730 households from 321 EAs in 13 provinces were sampled for IFLS. The detail on IFLS data and supporting

For peer review only - http://bmjopen.bmj.com/site/about/guidelines.xhtml

documents such as the survey protocol and questionnaires are publicly accessible through RAND's website.[24] The IFLS5 was approved by the relevant ethical review committees in the USA and Indonesia.

In our study, we included 42,083 individuals aged 15 years or older who had complete data for all study variables (98.2% of the total sample). For the analysis of cardiovascular-related preventive care utilisation, we included 26,612 individuals aged 31 years or older, which is 89.9% of the total number of individuals aged 31 years or older in the sample (29,612 individuals) and 63.2% of the total all-age sample (42,083 individuals). The present study excluded respondents aged 31 years or older because the risk of cardiovascular diseases substantially increases only after the age of 30 years.

Measurements

The individual's educational level and income were used as indicators of socioeconomic status (SES). Educational level was defined according to the International Standard Classification of Education 2011 issued by UNESCO. Based on the highest level completed by each individual, educational level was categorised into pre-primary, primary, lower secondary, upper secondary and tertiary level.

The level of household consumption was used as a proxy of income. In developing countries, consumption is considered a valid direct measurement of income or household wealth.[25] This measured at household level counted food, non-food consumables, durable goods, spending on education, and housing. These counts were aggregated and transformed into a monthly consumption, which was adjusted for household size to consider the economics of scale.

We also adjusted for geographical differences in purchasing parity, using Jakarta's poverty line as a reference. Income measurement for different areas was adjusted taking into account variations in the poverty line by province, as well as urban vs. rural place of residence. Data on the poverty line were obtained from the Indonesian Central Bureau of Statistics.

Healthcare utilisation data as collected by the IFLS5 were used; we measured the utilisation of: i) (outpatient) primary care, ii) outpatient secondary care, iii) total outpatient care, iv) inpatient care, and

For peer review only - http://bmjopen.bmj.com/site/about/guidelines.xhtml

v) cardiovascular-related preventive care. Primary care included any visits to or visits by trained health personnel from a public primary care centre, private primary care clinic, and/or private primary care physician practice. Outpatient secondary care included any visit to a public hospital outpatient care (polyclinics) and private hospital outpatient care. The IFLS5 questionnaire measured all outpatient care that was received during a four-week reference period.

Inpatient care was defined as any use of inpatient care during the previous 12 months for medical purposes, irrespective of the length of hospital stay. This included any use of inpatient care at primary care level with inpatient facilities, at public hospitals, or private hospitals. For preventive care utilisation, we focused on cardiovascular diseases-related preventive care because of the sizeable contribution of cardiovascular diseases to the overall disease burden in Indonesia.[26] The use of cardiovascular risk factor screening was measured, including blood pressure measurements, cholesterol tests, blood glucose tests, and electrocardiography (ECG) tests during the previous 12 months.

As a proxy of healthcare need, self-assessed health (SAH) was used. SAH is regarded as a health status measurement applicable to different socioeconomic groups. Data on SAH measurement from the IFLS5 survey were used, in which SAH was measured by asking “In general, how is your health?”; the four response categories were “very healthy”, “somewhat healthy”, “somewhat unhealthy”, and “very unhealthy.

Data analysis

To describe variation in healthcare use among socioeconomic groups, while taking into account differences between these groups in the age and sex structure, we calculated standardised prevalence rate (SPR) for each type of healthcare utilisation by educational levels and income quintiles. SPR was calculated as the number of cases per 100 persons and was standardised by age and sex the direct method, with the total survey population as the standard population. Next, the rate difference and the rate ratio were calculated based on the SPR of the two lowest SES groups combined, and the two highest

For peer review only - http://bmjopen.bmj.com/site/about/guidelines.xhtml

SES groups combined, respectively. These SES groups were combined to provide a more stable estimation of the rate difference and the rate ratio between the lower and higher SES groups, respectively. [27] It complements the RII, as the latter takes into account all SES groups separately.

The relative index inequality (RII) was used to estimate the magnitude of inequalities in healthcare utilisation in a more comprehensive way. The RII is a regression-based index that assesses the probability of healthcare use in relationship to the relative hierarchical position of every individual within the socioeconomic hierarchy. We assigned the fractional rank of the socioeconomic indicators (income and educational) as the main predictor in the logistic regression model (considering the binary outcome of outpatient and inpatient care utilisation). The RII was obtained from the value of odds ratio (OR) from the fractional rank of the socioeconomic indicators. The regression model was adjusted for age, sex and healthcare need, by controlling for SAH in the final model. Details on how RII calculated can be found elsewhere.[28]

A higher RII indicates a stronger association between this hierarchical position and healthcare utilisation and implies a greater difference in utilisation between higher SES groups compared to lower SES groups. More specifically, RII=1 indicates equality, RII<1 indicates higher utilisation among lower SES, and RII>1 indicates higher utilisation among higher SES. The RII was chosen because it commonly used in epidemiological research and has relatively a straightforward interpretation for readers who have no economics background compared to other common inequality measurements such as concentration index.

To correct for attrition and oversampling, the study sample was weighted with individual weights provided by the IFLS5. We used IBM SPPS Statistics 24 as a statistical package to analyse the data.

Patient and public involvement

No patients were involved in this study; members of the public were not directly involved in this study.

For peer review only - http://bmjopen.bmj.com/site/about/guidelines.xhtml

RESULTS

The study sample included slightly more female respondents (51.6%) than males (Table 1). Almost two-thirds of the respondents were aged 15-45 years. Males had a generally higher level of education as compared to females. Primary care was the most frequently used type of healthcare, with 14.6% of the respondents reporting that they utilised primary care at least once in the previous four weeks. The highest utilisation of preventive care was for blood pressure measurement, with 80.5% of the respondents reporting that their blood pressure was measured during the previous 12 months.

The prevalence rates of primary care use were about similar across all educational levels (Table 2, see also online supplementary file Figure 1). Outpatient secondary care utilisation was more frequent among people with a higher educational level compared to people with a lower educational level. For overall inpatient care utilisation, the prevalence rates gradually increased with increasing educational level. A linear association was found between healthcare utilisation and income quintiles for all types of healthcare. This association was particularly strong for utilisation of outpatient secondary care and inpatient care.

Table 3 (see also online supplementary file Figures 2 and 3) quantifies the magnitude of educational and income inequalities in the utilisation of healthcare. Our findings from simple inequality measurement (rate ratio and rate difference) showed similarities with the findings from sophisticated inequality measurement (RII). No educational inequalities were found in primary care utilisation in the crude analysis, but positive educational inequalities (i.e. higher education associated with higher use rates) emerged after adjusting for SAH (RII 1.13, 95% CI 1.01-1.26). We consistently found positive educational inequalities in all types and levels of health care use after adjusting for SAH. The largest educational inequality was found in outpatient secondary care utilisation (RII 10.35, 95% CI 8.11-13.22).

Positive income inequalities (i.e. higher income associated with higher use rates) were found in all types and levels of healthcare use, especially after adjustment for SAH. Similar to educational

For peer review only - http://bmjopen.bmj.com/site/about/guidelines.xhtml

inequalities, the largest income inequality was found in outpatient secondary care utilisation (RII 7.43, 95% CI 5.88-9.39). Generally, larger inequalities were found in relationship to income as compared to educational level, except for utilisation of outpatient secondary care.

A consistent linear association was found between prevalence rate of preventive care utilisation and SES (Table 4, and online supplementary file Figure 1). The prevalence rate of blood pressure measurement increased incrementally by SES group for both educational level and income quintiles. The prevalence rate of cholesterol tests, blood glucose tests and ECG tests drastically increased from the third highest SES groups to the highest SES groups, both for income and educational level. The differences were larger in relationship to educational level than to income.

Table 5 shows the estimates of the size socioeconomic inequalities in preventive care utilisation (see also online supplementary file Figures 2 and 3). Our analyses showed consistent findings between simple (rate difference and rate ratio) and sophisticated inequality estimations (RII). Exceptionally large positive educational inequalities were found in blood glucose tests (RII 30.31, 95% CI 26.13-35.15) and ECG tests (RII 30.90, 95% CI 24.97-38.23). For income inequalities, inequalities in preventive care utilisation were smaller compared to educational inequalities. ECG tests showed the largest income inequality (RII 12.96, 95% CI 10.68-15.73), and blood pressure measurements showed the smallest inequality (RII 3.40, 95% CI 3.04-3.79).

DISCUSSION

This study documented socioeconomic inequalities in healthcare utilisation among the adult population in Indonesia. These inequalities were particularly large for secondary and preventive care. Compared to educational inequalities, income-related inequalities were larger for primary care and inpatient care, but smaller for outpatient secondary and preventive care.

For peer review only - http://bmjopen.bmj.com/site/about/guidelines.xhtml

For peer review only

This study was based on a nationally representative survey with a high response rate (95.3%) and with measurements that matched established international standards.[29] A possible limitation of the study is the measurement of healthcare need, that was limited to SAH. Ideally, we would have used multiple measures of healthcare need, such as self-reported morbidities or health functioning. Although our dataset provided self-reported morbidities and data on health functioning, these are likely to be underestimated in the Indonesian population (particularly in lower SES groups)[30], and therefore invalid for healthcare need adjustments.

Because no registry-based data on inequalities in healthcare utilisation in Indonesia are available, we used self-reported use of healthcare. Such healthcare utilisation measures may be subject to recall bias. However, the problem of recall bias might be limited, as the prevalence values of outpatient and inpatient care utilisation from the IFLS5 are close to the national average in Indonesia as reported by the Ministry of Health and data from the National Economic Survey.[31 32]

Previous studies in Indonesia mostly focus on specific healthcare services such as maternal and child-related healthcare. Our findings show that the direction and magnitude of inequalities in healthcare use among individuals aged 15 years or older bear a resemblance to the large socioeconomic inequalities in maternal healthcare and child healthcare.[18 22] Similar to the recent study on wealth-related inequality in healthcare utilisation in Indonesia, we found smaller inequalities in the utilisation of primary care, especially outpatient care, and larger inequalities in secondary care.[23] Our results are also consistent with studies performed in other LMICs showing relatively small inequalities in PC utilisation, and larger inequalities in secondary care.[7-10]

The small socioeconomic inequalities in primary care utilisation are probably related to the relatively high supply and geographical distribution of primary care providers in Indonesia. Of all registered physicians in Indonesia, 78.4% are general practitioners that mostly practice as public or private providers. In total, there are 9745 public primary care centres providing services for the national

For peer review only - http://bmjopen.bmj.com/site/about/guidelines.xhtml

population with subsidy by local governments.[33 13] Moreover, according to recent studies, access to primary care was increased by a government-financed NHI program that aimed to reduce financial barriers of the poor population to healthcare.[32 34 23] In the NHI program, primary care acted as gatekeeper which required all the beneficiaries regardless of their socioeconomic background (poor people or government employee) to use primary care as an entry point to access the healthcare service.[35] For people without insurance coverage, primary care is relatively affordable and can be accessed at low cost, even in private practices.[13] This likely explained the smaller income and educational-related inequalities in the primary care utilisation compared to the inequalities in secondary and inpatient care utilisation.

In contrast to primary care, the use of secondary care facilities in Indonesia showed considerable inequalities by both educational level and income. For example, individuals with the highest income had seven times higher odds to use outpatient secondary care compared to those with the lowest income. It is likely that geographical barriers contribute to these inequalities. Because most secondary care facilities and specialists are located in urban areas, the poor need to pay high indirect costs (in terms of travel and opportunity) to access secondary care, even if their medical costs are covered by the NHI program.[23 32 34] Moreover, there is a limited supply of secondary care specialists; these specialists tend to work in private for-profit healthcare providers, which are not contracted by the NHI program. This is likely to result in low financial access for lower SES groups rather than higher SES groups, which may have supplementary private health insurance.[18 21]

We observed inequalities to be larger outpatient secondary care than for inpatient care. A possible explanation is that outpatient secondary care is much more affordable for higher income groups than for lower groups, as the former can pay the service by out-of pocket payment or private health insurance. Lower income groups generally can use outpatient secondary care only by using government health insurance with its referral system. For inpatient care, however, utilisation costs are significant for

For peer review only

For peer review only - http://bmjopen.bmj.com/site/about/guidelines.xhtml

higher income groups as well as lower income groups, and usually only affordable via government health insurance and accessible through a referral system.[21]

Inefficient referral procedures could also have contributed to larger inequalities in secondary care utilisation compared to primary care, particularly for educational-related inequalities. Even when low-educated people are entitled to access secondary healthcare, they may lack the knowledge required to obtain a referral, due to the complexity of the administrative procedures in the referral system.[34] Inequalities in secondary care may also be influenced by differences between educational groups in the preferences and resources that influence the way people utilise healthcare.[36] An Indonesian study showed that patients with higher educational level, regardless of their income level, were more likely to judge the quality of primary care to be low and to ask for a referral to secondary care. This tendency was not observed among people with high income, but relatively low education.[37 38] Education-related preferences might explain why educational inequalities in outpatient secondary care were larger compared to income-related inequalities.

We observed exceptionally large socioeconomic inequalities in preventive care, particularly by education. For example, individuals who had the highest educational level had 30 times higher odds to have a blood glucose test in the previous 12 months compared to those who had the lowest educational level. The individual's level of health literacy may play a major role in their use of preventive care.[39] Those with a relatively low level of health literacy may experience cognitive barriers to make decisions regarding diagnostic tests and treatments that they may need, irrespective of financial, geographic or administrative barriers.[40 41] This also likely explains relatively smaller educational-related inequalities in blood pressure measurement compared to other types of preventive care because blood pressure disorder such as high blood pressure is relatively known by common people regardless their educational background compared to other types of preventive care

For peer review only - http://bmjopen.bmj.com/site/about/guidelines.xhtml

The exceptionally large inequalities in preventive care utilisation may reflect the low priority given to preventive care in Indonesia's health policy which to date has strongly focused on curative care. This resulted in low health expenditures on preventive care[42], and the absence of a nationwide preventive program for the NCDs. As a result, the utilisation of preventive care is relying more on personal resources or potentially motivated or initiated by physicians who have more attention to preventive care.[43 44]

CONCLUSIONS

The findings underline the need to develop comprehensive efforts to tackle significant socioeconomic inequalities in healthcare utilisation in Indonesia. Potential areas of priority include removing financial and geographical barriers by providing the NHI program with universal health coverage, improving the supply and distribution of secondary care services, simplifying the referral system procedure, and developing a nationwide preventive care program. Improving the quality of primary care by providing better infrastructure and developing the competence of health personnel may have large impact on population health considering the (equality in) accessibility of primary care, and could potentially reduce the burden of secondary care. Monitoring healthcare (in)equality will be essential to evaluate the impact of these policies. Further research is needed to assess inequalities in healthcare among specific patient groups, and to evaluate the contribution of patient preferences and resources, and to examine the role of geographical factors and healthcare organisation and infrastructure. Such in-depth analyses could provide a better understanding of socioeconomic inequalities in healthcare utilisation in Indonesia and guide the development of strategies to address those inequalities.

Acknowledgement The authors thank the RAND Corporation for providing the dataset of the Indonesian Family Life Survey (IFLS) for this study.

Author Contributions JM conceived the paper. JM and AEK developed the analysis strategy. JM conducted the data analysis. JM, DSK, AEK collectively interpreted the findings. JM prepared the initial

For peer review only - http://bmjopen.bmj.com/site/about/guidelines.xhtml

draft of the manuscript. JM, DSK, AEK equally contributed to the revision of the manuscript. All authors have read and approved the final manuscript.

Funding This study is funded by the Indonesian Endowment Fund for Education (LPDP), Ministry of Finance, Republic of Indonesia, grant number 20160322045795. The funding source has no role in study design, data collection, data analysis and interpretation, manuscript writing, and decision to submit or publish.

Competing Interest None declared

Patient consent Not required

Ethics approval This study is a secondary analysis using the Indonesian Family Life Survey (IFLS) dataset. The IFLS was approved by the Institutional Review Board (IRB) of the Rand Corporation (USA) and the Survey Meter (Indonesia). The data set is publicly available and no personal information can be identified. This study is categorised as being exempt from human research according to the National Institute of Health (NIH).

Provenance and peer review Not commissioned; externally peer reviewed.

Data sharing statement This study used the Indonesian Family Life Survey (IFLS) dataset provided by RAND Corp. The IFLS dataset (including the supporting documents such as survey protocol and questionnaire) is freely accessible at https://www.rand.org/labor/FLS/IFLS.html.

Open Access This is an Open Access article distributed in accordance with the Creative Commons Attribution Non Commercial (CC BY-NC 4.0) license, which permits others to distribute, remix, adapt, build upon this work non-commercially, and license their derivative works on different terms, provided the original work is properly cited and the use is non-commercial. See: http://creativecommons.org/licenses/by-nc/4.0/

REFERENCES

1. Oliver A, Mossialos E. Equity of access to health care: outlining the foundations for action. *Journal of epidemiology and community health* 2004;58(8):655-58.
2. Culyer AJ, Wagstaff A. Equity and equality in health and health care. *Journal of health economics* 1993;12(4):431-57.
3. Goddard M, Smith P. Equity of access to health care services: theory and evidence from the UK. *Soc Sci Med* 2001;53 doi: 10.1016/s0277-9536(00)00415-9
4. van Doorslaer E, Masseria C, Koolman X. Inequalities in access to medical care by income in developed countries. *Cmaj* 2006;174 doi: 10.1503/cmaj.050584
5. Walters S, Suhrcke M. Socioeconomic inequalities in health and health care access in central and eastern Europe and the CIS: a review of the recent literature. WHO European Office for Investment for Health and Development2005.
6. Balabanova D, McKee M, Pomerleau J. Health service utilization in the former soviet union: evidence from eight countries. *Health Serv Res* 2004;39 doi: 10.1111/j.1475-6773.2004.00326.x

For peer review only - http://bmjopen.bmj.com/site/about/guidelines.xhtml

7. Makinen M, Waters H, Rauch M, et al. Inequalities in health care use and expenditures: empirical data from eight developing countries and countries in transition. *Bulletin of the World Health Organization* 2000;78:55-65.
8. Zhang X, Wu Q, Shao Y, et al. Socioeconomic Inequities in Health Care Utilization in China. *Asia Pacific Journal of Public Health* 2015;27(4):429-38. doi: doi:10.1177/1010539514565446
9. Brinda EM, Attermann J, Gerdtham UG, et al. Socio-economic inequalities in health and health service use among older adults in India: results from the WHO Study on Global AGEing and adult health survey. *Public Health* 2016;141:32-41. doi: 10.1016/j.puhe.2016.08.005 [published Online First: 2016/12/10]
10. Yiengprugsawan V, Carmichael G, Lim LY, et al. Explanation of inequality in utilization of ambulatory care before and after universal health insurance in Thailand. *Health policy and planning* 2011;26(2):105-14. doi: 10.1093/heapol/czq028 [published Online First: 2010/08/26]
11. O'Donnell O. Access to health care in developing countries: breaking down demand side barriers. *Cadernos de saude publica* 2007;23(12):2820-34.
12. Peters DH, Garg A, Bloom G, et al. Poverty and access to health care in developing countries. *Annals of the New York Academy of Sciences* 2008;1136:161-71. doi: 10.1196/annals.1425.011
13. Mahendradhata Y, Trisnantoro L, Listyadewi S, et al. The Republic of Indonesia Health System Review. In: Hort K, Patcharanarumol W, eds. Health Systems in Transition. India: World Health Organization, Regional Office for South-East Asia, 2017.
14. Mboi N. Indonesia: On the Way to Universal Health Care. *Health Systems & Reform* 2015;1(2):91-97. doi: 10.1080/23288604.2015.1020642
15. Pisani E, Olivier Kok M, Nugroho K. Indonesia's road to universal health coverage: a political journey. *Health policy and planning* 2017;32(2):267-76. doi: 10.1093/heapol/czw120
16. Agustina R, Dartanto T, Sitompul R, et al. Universal health coverage in Indonesia: concept, progress, and challenges. *The Lancet* 2019;393(10166):75-102. doi: 10.1016/S0140-6736(18)31647-7
17. Widjaja FF. Universal health coverage in Indonesia – the forgotten prevention. *2014* 2014;23(3):-118 %J Medical Journal of Indonesia. doi: 10.13181/mji.v23i3.1070 [published Online First: 2014-10-22]
18. Barber SL, Gertler PJ, Harimurti P. Differences In Access To High-Quality Outpatient Care In Indonesia. *Health Affairs* 2007;26(3):w352-w66. doi: 10.1377/hlthaff.26.3.w352
19. Maharani DA. Inequity in dental care utilization in the Indonesian population with a self-assessed need for dental treatment. *The Tohoku journal of experimental medicine* 2009;218(3):229-39. [published Online First: 2009/06/30]
20. Maharani DA, Rahardjo A. Is the utilisation of dental care based on need or socioeconomic status? A study of dental care in Indonesia from 1999 to 2009. *International dental journal* 2012;62(2):90-4. doi: 10.1111/j.1875-595X.2011.00095.x [published Online First: 2012/03/17]
21. Utomo B, Sucahya PK, Utami FR. Priorities and realities: addressing the rich-poor gaps in health status and service access in Indonesia. *International Journal for Equity in Health* 2011;10(1):47. doi: 10.1186/1475-9276-10-47
22. Hosseinpoor AR, Bergen N, Floranita R, et al. State of health inequality: Indonesia. Geneva: World Health Organization, 2017.
23. Johar M, Soewondo P, Pujisubekti R, et al. Inequality in access to health care, health insurance and the role of supply factors. *Social Science & Medicine* 2018;213:134-45. doi: https://doi.org/10.1016/j.socscimed.2018.07.044
24. Strauss J, Witoelar F, Sikoki B. The Fifth Wave of the Indonesia Family Life Survey: Overview and Field Report: RAND Labor and Population, 2016.
25. O'Donnel O, Doorslaer Ev, Wagstaff A, et al. Analyzing Health Equity Using Household Survey Data: A Guide to Techniques and Their Implementation. Washington: The World Bank Institute 2008.

For peer review only - http://bmjopen.bmj.com/site/about/guidelines.xhtml

26. Riley LM, Cowan M. Noncommunicable Diseases Country Profile 2014. Geneva: World Health Organization, 2014.
27. Hosseinpoor AR, Bergen N. Handbook on Health Inequality Monitoring with a special focus in low-middle income countries Geneva: Worrld Health Organization, 2013.
28. Mackenbach JP, Kunst AE. Measuring the magnitude of socio-economic inequalities in health: an overview of available measures illustrated with two examples from Europe. *Soc Sci Med* 1997;44(6):757-71.
29. Galobardes B, Lynch J, Smith GD. Measuring socioeconomic position in health research. *British Medical Bulletin* 2007;81-82(1):21-37. doi: 10.1093/bmb/ldm001
30. Vellakkal S, Millett C, Basu S, et al. Are estimates of socioeconomic inequalities in chronic disease artefactually narrowed by self-reported measures of prevalence in low-income and middle-income countries? Findings from the WHO-SAGE survey. *Journal of epidemiology and community health* 2014:jech-2014-204621.
31. Riset Kesehatan Dasar 2013. Jakarta: Badan Penelitian dan Pengembangan Kementerian Kesehatan Republik Indonesia 2013.
32. Sparrow R, Suryahadi A, Widyanti W. Social health insurance for the poor: Targeting and impact of Indonesia's Askeskin programme. *Social Science & Medicine* 2013;96:264-71. doi: http://doi.org/10.1016/j.socscimed.2012.09.043
33. Profil Kesehatan Indonesia 2015. Jakarta: Kementerian Kesehatan Republik Indonesia, 2016.
34. Harimurti P, Pambudi E, Pigazzini A, et al. The Nuts & Bolts of Jamkesmas, Indonesia's Government-financed Health Coverage Program for the Poor and Near-Poor. UNICO Studies Series. Washington DC: The World Bank, 2013.
35. Hidayat B, Thabrany H, Dong H, et al. The effects of mandatory health insurance on equity in access to outpatient care in Indonesia. *Health policy and planning* 2004;19(5):322-35. doi: 10.1093/heapol/czh037
36. Levesque J-F, Harris MF, Russell G. Patient-centred access to health care: conceptualising access at the interface of health systems and populations. *International Journal for Equity in Health* 2013;12:18-18. doi: 10.1186/1475-9276-12-18
37. Istiono W, Claramita M, Ekawati FM, et al. Physician's self-perceived abilities at primary care settings in Indonesia. *Journal of Family Medicine and Primary Care* 2015;4(4):551-58. doi: 10.4103/2249-4863.174286
38. Sari MI, Prabandari YS, Claramita M. Physicians' professionalism at primary care facilities from patients' perspective: The importance of doctors' communication skills. *Journal of Family Medicine and Primary Care* 2016;5(1):56-60. doi: 10.4103/2249-4863.184624
39. Gottfredson LS. Intelligence: is it the epidemiologists' elusive" fundamental cause" of social class inequalities in health? *Journal of personality and social psychology* 2004;86(1):174.
40. Chen JZ, Hsu HC, Tung HJ, et al. Effects of health literacy to self-efficacy and preventive care utilization among older adults. *Geriatrics & gerontology international* 2013;13(1):70-6. doi: 10.1111/j.1447-0594.2012.00862.x [published Online First: 2012/06/08]
41. Huang C, Liu CJ, Pan XF, et al. Correlates of unequal access to preventive care in China: a multilevel analysis of national data from the 2011 China Health and Nutrition Survey. *BMC Health Serv Res* 2016;16:177. doi: 10.1186/s12913-016-1426-2 [published Online First: 2016/05/14]
42. Widjaja FF. Universal health coverage in Indonesia–the forgotten prevention. *Med J Indones* 2014;23(3)
43. Rodin D, Stirbu I, Ekholm O, et al. Educational inequalities in blood pressure and cholesterol screening in nine European countries. *J Epidemiol Community Health* 2012;66(11):1050-5. doi: 10.1136/jech-2011-200273 [published Online First: 2012/01/17]

For peer review only - http://bmjopen.bmj.com/site/about/guidelines.xhtml

44. Palència L, Espelt A, Rodríguez-Sanz M, et al. Socio-economic inequalities in breast and cervical cancer screening practices in Europe: influence of the type of screening program. *International Journal of Epidemiology* 2010;39(3):757-65. doi: 10.1093/ije/dyq003

For peer review only

For peer review only - http://bmjopen.bmj.com/site/about/guidelines.xhtml

Table 1. Basic characteristics of the study population.

Variables	Total		Male		Female	
	n	%	n	%	n	%
**Gender**						
Male	20374	48.4	-	-	-	-
Female	21709	51.6	-	-	-	-
**Age group (in years)**						
15-30	12471	29.6	6436	31.6	6035	27.8
31-45	14049	33.4	6545	32.1	7503	34.6
46-60	10280	24.4	4973	24.4	5306	24.4
>60	5283	12.6	2419	11.9	2864	13.2
**Education level**						
Pre-primary	9868	23.4	3977	19.5	5891	27.1
Primary	9993	23.7	4855	23.8	5138	23.7
Lower secondary	8082	19.2	4041	19.8	4041	18.6
Upper secondary	10731	25.2	5894	28.9	4838	22.3
Tertiary	3409	8.1	1607	7.9	1802	8.3
**Income[a]**						
1st quintile (230-1300)	8417	20.0	4050	19.9	4367	20.1
2nd quintile (1300-1830)	8418	20.0	3997	19.6	4421	20.4
3rd quintile (1830-2520)	8415	20.0	4056	19.9	4359	20.1
4th quintile (2520-3830)	8417	20.0	4159	20.4	4258	19.6
5th quintile (3830-55400)	8416	20.0	4111	20.2	4305	19.8
**Self-assessed health**						
Very healthy	8137	19.3	4362	21.4	3775	17.4
Somewhat healthy	24757	58.8	12179	59.8	12578	57.9
Somewhat unhealthy	8447	20.1	3513	17.2	4934	22.7
Very unhealthy	742	1.8	320	1.6	422	1.9
**Outpatient care utilisation**						
Primary care	6155	14.6	2006	9.8	4149	19.1
Secondary care	1022	2.4	427	2.1	595	2.7
Total	6864	16.3	2323	11.4	4541	20.9
**Inpatient care utilisation**						
Overall	1937	4.6	591	2.9	1346	6.2
**Preventive care utilisation (age ≥ 31 years)**						
Blood pressure screening	21663	80.5	9254	74.3	12409	85.4
Cholesterol screening	4678	17.4	1951	15.7	2727	18.9
Blood glucose screening	4142	15.4	1855	14.9	2287	15.8
Electrocardiography test	1723	6.4	876	7.0	847	5.9

[a]Income in thousands Indonesian Rupiah (IDR)

For peer review only - http://bmjopen.bmj.com/site/about/guidelines.xhtml

Table 2. Standardised prevalence rate (SPR) of healthcare utilisation by socioeconomic status.

	Outpatient care (SPR, 95% CI)†			Inpatient care (SPR, 95% CI)
	**Primary**	**Secondary**	**Total**	**Overall**
**Education**				
Pre-primary	14.47(13.78-15.18)	1.31(1.12-1.53)	15.22(14.52-15.95)	3.07(2.77-3.40)
Primary	14.93(14.19-15.70)	2.03(1.76-2.32)	16.07(15.30-16.87)	4.60(4.18-5.04)
Lower secondary	15.00(14.12-15.91)	1.88(1.57-2.22)	16.38(15.47-17.33)	4.31(3.86-4.81)
Upper secondary	14.38(13.64-15.16)	3.39(3.03-3.79)	16.99(16.18-17.84)	6.12(5.63-6.64)
Tertiary	14.37(13.12-15.71)	6.26(5.44-7.17)	18.97(17.53-20.50)	6.93(6.04-7.91)
**Income**				
1st quintile	11.71(11.02-12.42)	1.32(1.09-1.57)	12.54(11.83-13.29)	2.99(2.65-3.35)
2nd quintile	14.11(13.34-14.92)	1.44(1.20-1.71)	14.73(13.94-15.55)	3.76(3.37-4.18)
3rd quintile	15.36(14.54-16.22)	2.00(1.71-2.33)	16.75(15.89-17.64)	4.24(3.81-4.70)
4th quintile	15.73(14.88-16.62)	2.91(2.55-3.31)	18.57(17.64-19.53)	5.13(4.64-5.65)
5th quintile	16.13(15.24-17.06)	4.97(4.48-5.50)	19.83(18.74-20.85)	7.56(6.94-8.21)

†Prevalence rate per 100 persons, age and sex standardised to the total population.

Table 3. Socioeconomic inequalities in the utilisation of various types and levels of healthcare.

	Type of care	Level of care	SPR (95% CI)†		Rate difference	Rate ratio	RII (95% CI), adjusted for age, sex	RII (95% CI), adjusted for age, sex, SAH
			Two lowest groups	Two highest groups				
Education	Outpatient	Primary	14.68(14.18-15.20)	14.38(13.74-15.05)	-0.30	0.98	0.99 (0.98-1.01)	1.13 (1.01-1.26)
		Secondary	1.64(1.47-1.82)	2.88 (2.59-3.19)	1.24	1.76	7.89 (6.33-9.85)	10.35 (8.11-13.22)
		Total	15.62(15.09-16.15)	17.51(16.80-18.25)	1.89	1.12	1.35 (1.24-1.46)	1.59 (1.44-1.77)
	Inpatient	Overall	3.74(3.48-4.01)	6.31(5.88-6.76)	2.57	1.69	2.38 (1.97-2.76)	2.78 (2.32-3.32)
Income	Outpatient	Primary	12.88(12.35-13.42)	16.25(15.62-16.89)	3.37	1.26	1.50 (1.39-1.62)	1.68 (1.52-1.85)
		Secondary	1.36(1.20-1.54)	4.69(4.35-5.04)	3.33	3.45	6.61 (5.29-8.25)	7.43 (5.88-9.39)
		Total	13.61(13.08-14.16)	19.18(18.50-19.88)	5.57	1.41	1.80 (1.67-1.94)	2.15 (1.96-2.36)
	Inpatient	Overall	3.36(3.10-3.63)	6.30(5.91-6.71)	2.94	1.88	2.94 (2.52-3.43)	3.11 (2.63-3.66)

†Prevalence rate per 100 persons, age and sex standardised to the total population.

For peer review only

For peer review only - http://bmjopen.bmj.com/site/about/guidelines.xhtml

Table 4. Standardised prevalence rate (SPR) of preventive care utilisation by socioeconomic status.

	Preventive care activity (SPR, 95% CI)†			
	Blood pressure	Cholesterol	Blood glucose	ECG
**Education**				
Pre-primary	72.40(70.54-74.30)	8.85(8.24-9.49)	6.85(6.32-7.41)	2.51(2.19-2.86)
Primary	79.54(77.47-81.65)	10.78(10.03-11.58)	10.59(9.84-11.39)	4.00(3.54-4.50)
Lower secondary	82.72(79.88-85.63)	18.03(16.67-19.48)	15.44(14.17-16.79)	4.95(4.25-5.73)
Upper secondary	86.66(84.27-89.10)	26.83(25.44-28.27)	24.72(23.39-26.10)	10.80(9.94-11.71)
Tertiary	92.33(88.45-96.35)	44.72(41.97-47.60)	43.38(40.66-46.23)	20.99(19.13-22.99)
**Income**				
1st quintile	73.55(71.27-75.88)	7.83(7.11-8.60)	6.12(5.49-6.81)	2.71(2.29-3.18)
2nd quintile	76.77(74.45-79.15)	12.12(11.21-13.08)	10.14(9.30-11.02)	3.37(2.90-3.90)
3rd quintile	80.60(78.22-83.03)	14.37(13.37-15.42)	12.63(11.70-13.63)	4.64(4.08-5.26)
4th quintile	83.68(81.25-86.16)	20.34(19.14-21.59)	18.41(17.27-19.60)	7.13(6.43-7.89)
5th quintile	87.87(85.38-90.41)	32.69(31.17-34.26)	30.09(28.64-31.60)	14.43(13.43-15.49)

†Prevalence rate per 100 persons, age and sex standardised to the total population

Table 5. Socioeconomic inequalities in the use of various preventive care activities.

	Activity	SPR (95% CI)†		Rate difference	Rate ratio	RII (95% CI) adjusted for age, sex	RII (95% CI) adjusted for age, sex, SAH
		Two lowest groups	Two highest groups				
**Education**	Blood pressure	75.77(74.38-77.17)	88.28(86.24-90.36)	12.51	1.17	6.37 (5.61-7.24)	6.67(5.87-7.59)
	Cholesterol	9.69(9.22-10.19)	32.12(30.85-33.44)	22.43	3.31	18.17(15.91-20.74)	21.27(18.53-24.41)
	Blood glucose	8.47(8.03-8.94)	30.21(28.98-31.49)	21.74	3.57	24.61(21.36-28.36)	30.31(26.13-35.15)
	ECG	3.17(2.90-3.46)	13.74(12.92-14.60)	10.57	4.33	25.45(20.72-31.20)	30.90(24.97-38.23)
**Income**	Blood pressure	75.17(73.54-76.83)	86.28(84.54-88.05)	11.11	1.15	3.34(2.99-3.72)	3.40(3.04-3.79)
	Cholesterol	8.61(8.06-9.17)	26.54(25.57-25.53)	17.93	3.08	9.20(8.15-10.40)	9.76 (8.63-11.02)
	Blood glucose	8.11(7.59-8.66)	24.29(23.36-35.54)	16.18	3.00	10.81(9.49-12.30)	11.59(10.18-13.20)
	ECG	3.03(2.71-3.37)	10.78(10.14-11.40)	7.73	3.55	12.42(10.23-15.07)	12.96(10.68-15.73)

†Prevalence rate per 100 persons, age and sex standardised to the total population

For peer review only

For peer review only - http://bmjopen.bmj.com/site/about/guidelines.xhtml

Table 1. Distribution of self-assessed health status (SAH) among different socioeconomic status (SES)

	Self-assessed health (SAH)			
	Very healthy n (%)	Somewhat healthy n (%)	Somewhat unhealthy n (%)	Very unhealthy n (%)
**Educational level**				
Pre-primary	1900 (19.3)	4922 (49.9)	2693 (27.3)	353 (3.6)
Primary	1980 (19.8)	5652 (56.6)	2178 (21.8)	182 (1.8)
Lower secondary	1538 (19.0)	4956 (61.3)	1499 (18.5)	89 (1.1)
Upper secondary	2044 (19.0)	6957 (64.8)	1641 (15.3)	90 (0.8)
Tertiary	675 (19.8)	2270 (66.6)	436 (12.8)	27 (0.8)
**Income**				
1st quintile (poorest)	1683 (18.8)	5031 (56.3)	2036 (22.8)	194 (2.2)
2nd quintile	1637 (18.9)	5077 (58.5)	1778 (20.5)	188 (2.2)
3rd quintile	1649 (19.4)	5081 (59.8)	1619 (19.1)	143(1.7)
4th quintile	1622 (19.8)	4894 (59.7)	1570 (19.2)	112 (1.4)
5th quintile (richest)	1544 (19.9)	4674 (60.2)	1444 (18.6)	105 (1.4)

For peer review only

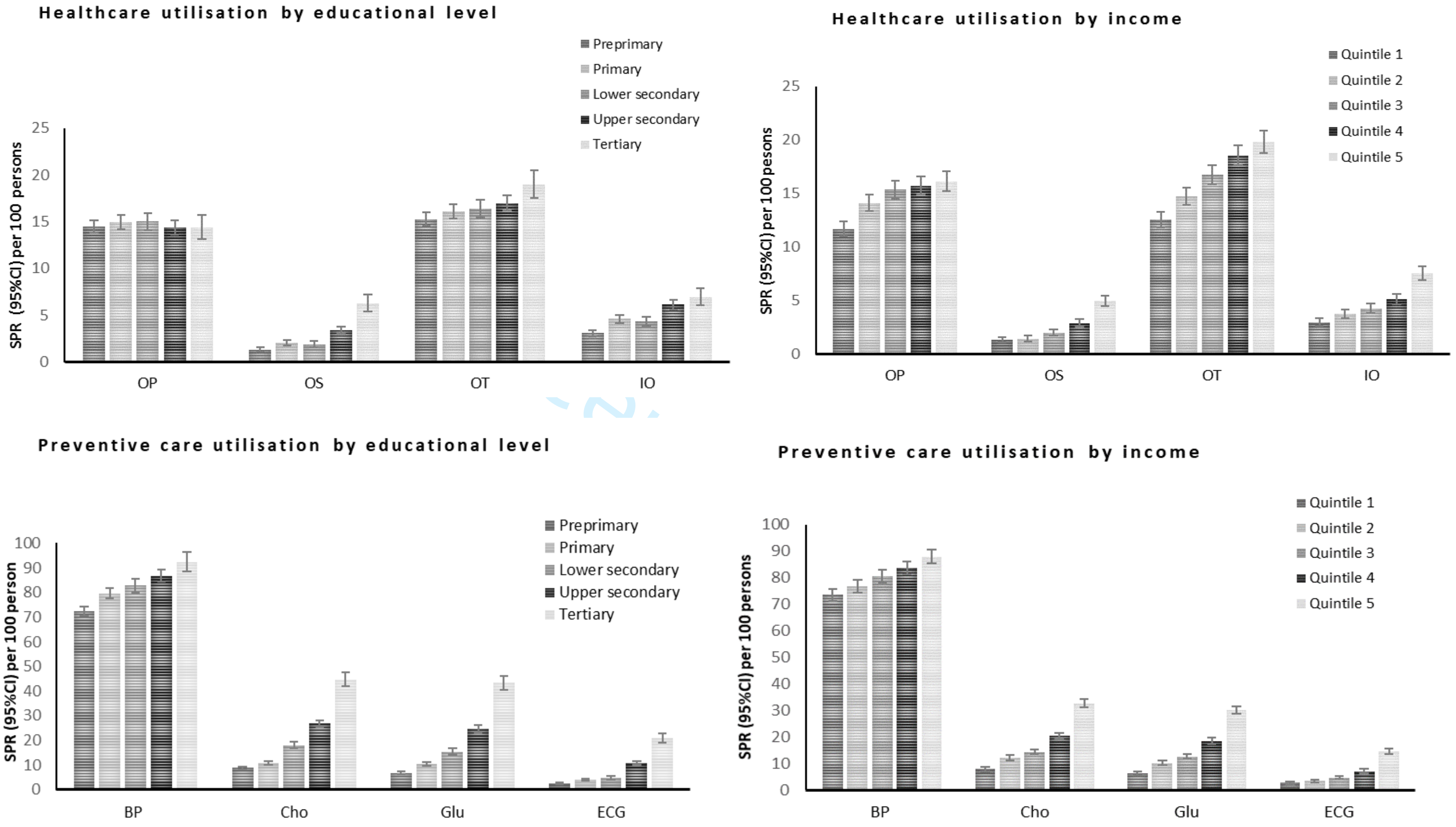

Figure 1. Standardised prevalence rate (95%CI) for healthcare and preventive care utilisation. Prevalence rate is per 100 persons, standardised by age and sex to total population. OP: Outpatient primary care; OS: Outpatient secondary care; OT: Outpatient total; IO: Inpatient overall; BP: Blood pressure; Cho: Cholesterol; Glu: Blood glucose; ECG: Electrocardiograph.

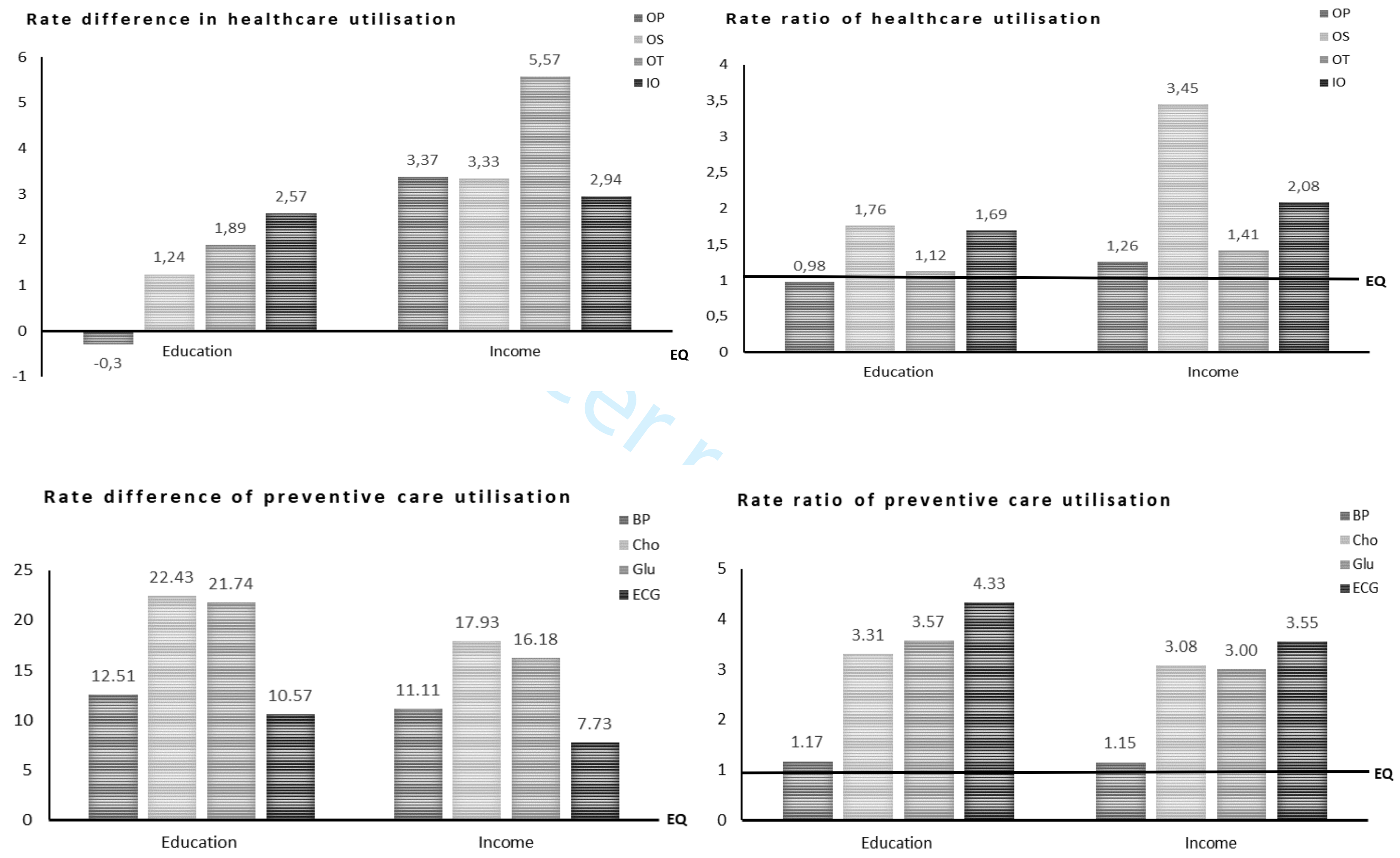

Figure 2. **Simple measurement of absolute (rate difference) and relative (rate ratio) inequalities in healthcare and preventive care utilisation between two highest and two lowest groups of SES. Rate difference is per 100 persons. OP: Outpatient primary care; OS: Outpatient secondary care; OT: Outpatient total; IO: Inpatient overall; BP: Blood pressure; Cho: Cholesterol; Glu: Blood glucose; ECG: Electrocardiograph; EQ: Equality line.**

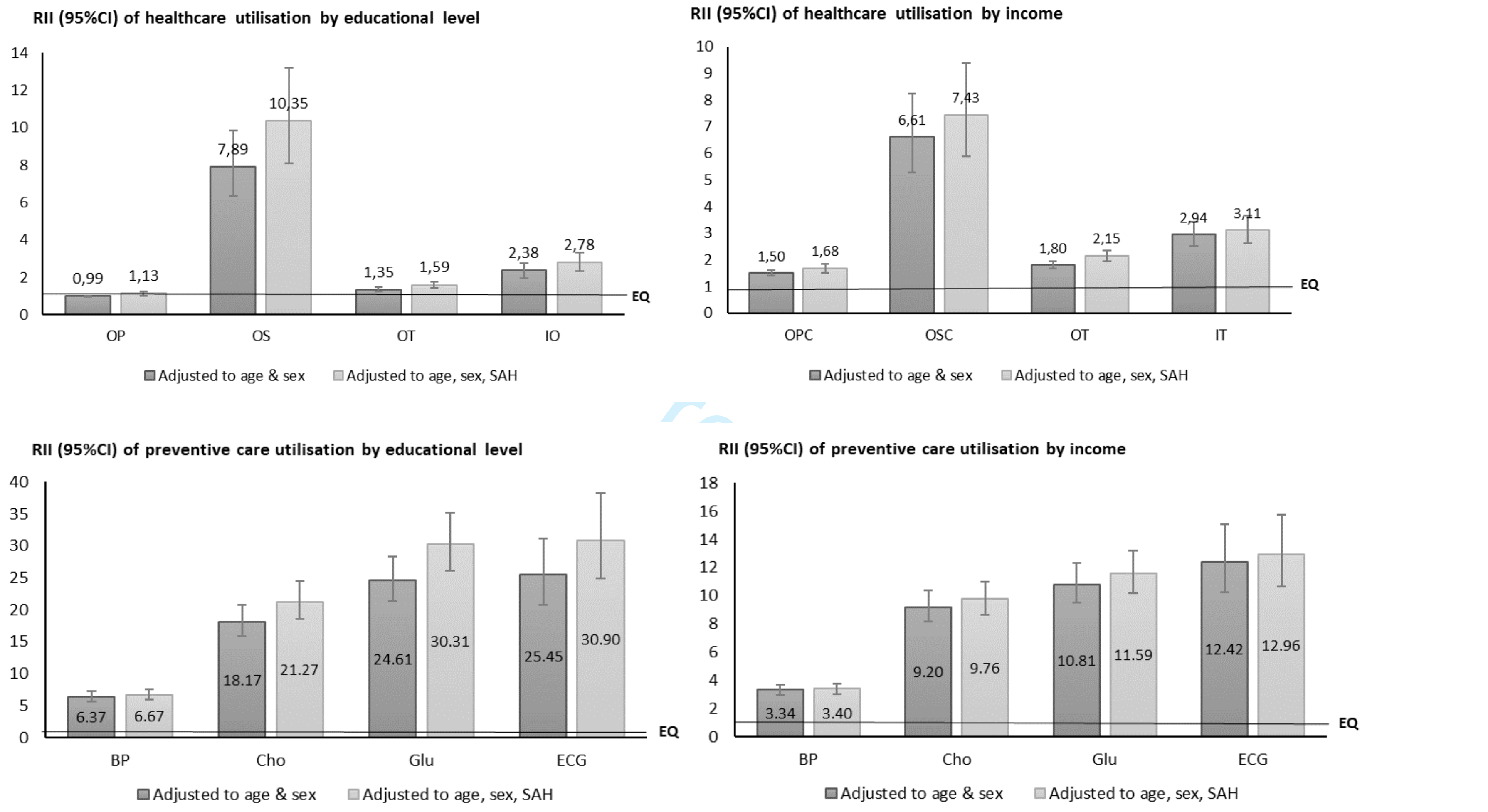

Figure 3. Relative index inequality (95%CI) of healthcare and preventive care utilisation by educational level and income. OP: Outpatient primary care; OS: Outpatient secondary care; OT: Outpatient total; IO: Inpatient overall; BP: Blood pressure; Cho: Cholesterol; Glu: Blood glucose; ECG: Electrocardiograph; EQ: Equality line; SAH: Self-assessed health

STROBE Statement—Checklist of items that should be included in reports of ***cross-sectional studies***

	Item No	Recommendation
**Title and abstract**	1	(*a*) Indicate the study's design with a commonly used term in the title or the abstract **[see methods section of abstract page 2]**
		(*b*) Provide in the abstract an informative and balanced summary of what was done and what was found **[see methods and results section of abstract page 2]**
**Introduction**		
Background/rationale	2	Explain the scientific background and rationale for the investigation being reported **[page 4-5]**
Objectives	3	State specific objectives, including any pre-specified hypotheses [**page 6]**
**Methods**		
Study design	4	Present key elements of study design early in the paper **[page 6]**
Setting	5	Describe the setting, locations, and relevant dates, including periods of recruitment, exposure, follow-up, and data collection **[page 6]**
Participants	6	(*a*) Give the eligibility criteria, and the sources and methods of selection of participants **[page 6]**
Variables	7	Clearly define all outcomes, exposures, predictors, potential confounders, and effect modifiers. Give diagnostic criteria, if applicable **[page 7]**
Data sources/ measurement	8*	For each variable of interest, give sources of data and details of methods of assessment (measurement). Describe comparability of assessment methods if there is more than one group **[page 7]**
Bias	9	Describe any efforts to address potential sources of bias **[page 6]**
Study size	10	Explain how the study size was arrived at **[page 6]**
Quantitative variables	11	Explain how quantitative variables were handled in the analyses. If applicable, describe which groupings were chosen and why **[page 7]**
Statistical methods	12	(*a*) Describe all statistical methods, including those used to control for confounding **[page 8-9]**
		(*b*) Describe any methods used to examine subgroups and interactions **[N/A]**
		(*c*) Explain how missing data were addressed **[page 5]**
		(*d*) If applicable, describe analytical methods taking account of sampling strategy **[page 9]**
		(*e*) Describe any sensitivity analyses **[N/A]**
**Results**		
Participants	13*	(a) Report numbers of individuals at each stage of study—eg numbers potentially eligible, examined for eligibility, confirmed eligible, included in the study, completing follow-up, and analysed **[N/A]**
		(b) Give reasons for non-participation at each stage **[N/A]**
		(c) Consider use of a flow diagram **[N/A]**
Descriptive data	14*	(a) Give characteristics of study participants (eg demographic, clinical, social) and information on exposures and potential confounders [**page 10, table 1 (page 20]**
		(b) Indicate number of participants with missing data for each variable of interest **[N/A]**
Outcome data	15*	Report numbers of outcome events or summary measures **[page 10-11, table 2,4 (page 21,22]**
Main results	16	(*a*) Give unadjusted estimates and, if applicable, confounder-adjusted estimates and their precision (eg, 95% confidence interval). Make clear which confounders

For peer review only

		were adjusted for and why they were included **[page 10-11, table 3,5 (page 21,22)]**
		(*b*) Report category boundaries when continuous variables were categorized **[N/A]**
		(*c*) If relevant, consider translating estimates of relative risk into absolute risk for a meaningful time period **[N/A]**
Other analyses	17	Report other analyses done—eg analyses of subgroups and interactions, and sensitivity analyses **[N/A]**
**Discussion**		
Key results	18	Summarise key results with reference to study objectives **[page 11]**
Limitations	19	Discuss limitations of the study, taking into account sources of potential bias or imprecision. Discuss both direction and magnitude of any potential bias **[page 12]**
Interpretation	20	Give a cautious overall interpretation of results considering objectives, limitations, multiplicity of analyses, results from similar studies, and other relevant evidence **[page 12-15]**
Generalisability	21	Discuss the generalisability (external validity) of the study results **[page 15]**
**Other information**		
Funding	22	Give the source of funding and the role of the funders for the present study and, if applicable, for the original study on which the present article is based **[declaration section, page 16]**

*Give information separately for exposed and unexposed groups.

Note: An Explanation and Elaboration article discusses each checklist item and gives methodological background and published examples of transparent reporting. The STROBE checklist is best used in conjunction with this article (freely available on the Web sites of PLoS Medicine at http://www.plosmedicine.org/, Annals of Internal Medicine at http://www.annals.org/, and Epidemiology at http://www.epidem.com/). Information on the STROBE Initiative is available at www.strobe-statement.org.

For peer review only

For peer review only - http://bmjopen.bmj.com/site/about/guidelines.xhtml
